# T Cell-Mediated Autoimmunity in Glaucoma Neurodegeneration

**DOI:** 10.3389/fimmu.2021.803485

**Published:** 2021-12-16

**Authors:** Lixiang Wang, Xin Wei

**Affiliations:** ^1^ Department of Ophthalmology, West China Hospital, Sichuan University, Chengdu, China; ^2^ Department of Ophthalmology, Shangjin Nanfu Hospital, Chengdu, China

**Keywords:** glaucoma, T cell, autoimmune, neurodegenerative disease, immune modulation therapy

## Abstract

Glaucoma as the leading neurodegenerative disease leads to blindness in 3.6 million people aged 50 years and older worldwide. For many decades, glaucoma therapy has primarily focused on controlling intraocular pressure (IOP) and sound evidence supports its role in delaying the progress of retinal ganglial cell (RGC) damage and protecting patients from vision loss. Meanwhile, accumulating data point to the immune-mediated attack of the neural retina as the underlying pathological process behind glaucoma that may come independent of raised IOP. Recently, some scholars have suggested autoimmune aspects in glaucoma, with autoreactive T cells mediating the chief pathogenic process. This autoimmune process, as well as the pathological features of glaucoma, largely overlaps with other neurodegenerative diseases in the central nervous system (CNS), including Alzheimer’s disease, Parkinson’s disease, and multiple sclerosis. In addition, immune modulation therapy, which is regarded as a potential solution for glaucoma, has been boosted in trials in some CNS neurodegenerative diseases. Thus, novel insights into the T cell-mediated immunity and treatment in CNS neurodegenerative diseases may serve as valuable inspirations for ophthalmologists. This review focuses on the role of T cell-mediated immunity in the pathogenesis of glaucoma and discusses potential applications of relevant findings of CNS neurodegenerative diseases in future glaucoma research.

## Introduction

The retina is an extension of the central nervous system (CNS) in the eye. Retinal ganglial cells (RGCs) are the primary sites of pathology in glaucoma (1). Their cell bodies reside in the inner retina, while their axes extend a long way through the optic nerve into the brain. Thus, glaucoma is considered a leading neurodegenerative disease that is estimated to affect 79.6 million people worldwide in 2020 (2). The pathogenic mechanisms of RGC loss in glaucoma are multifactorial, including elevated intraocular pressure (IOP), aging, oxidative stress, excitotoxicity, and mitochondrial dysfunction, but the full picture of glaucoma remains elusive due to its nature of high complexity and chronicity (3, 4). The role of immune-mediated neurodegeneration in glaucoma has been established in recent decades and is regarded as an important component in the pathogenesis of glaucoma.

Activation of residential immunocompetent cells in the retina (microglia and macroglia) and infiltration of peripheral immune cells (T cells, B cells, macrophages, monocytes, *etc.*) are found to be pathogenic and associated with the RGC loss (5, 6). Based on the discovery of autoantigens and self-reactive T cells, some scholars have proposed glaucoma to be an autoimmune disease with T cells playing a central role (7–9). Meanwhile, the essential roles of autoreactive T cells in the maintenance of tissue homeostasis and restriction of inflammation in the retina and CNS have also been elucidated in animal studies (10, 11). Evidence of either neurotoxic or neuroprotective roles of autoreactive T cells indicates the complexity of neuroinflammation in the progression of glaucoma. The role of autoreactive T cells is multifaceted, and an imbalance in T cell/microglia interactions is considered to be the culprit of glaucoma. Based on this, immunomodulation therapy has been proposed as a potential strategy for glaucoma (12).

As a representative of neurodegenerative disease in the eye, glaucoma shares many common pathological features with other CNS neurodegenerative disorders, including Alzheimer’s disease (AD), Parkinson’s disease (PD), and multiple sclerosis (MS) (13–15). Most importantly, they are all characterized by compromised barrier function and chronic neuroinflammation toward self-antigens (16–18). As the strategy to harness the immune response is most extensively explored in AD, it may provide valuable insights and experience for the development of a possible immune modulation therapy for glaucoma. In this review, we focus on the role of autoreactive T cells in glaucoma pathology and treatment options and systematically review publications in PubMed, Embase, and the Cochrane Library based on the topics of “glaucoma”, “T cell”, and “neurodegeneration”.

## Stress Response and Gut Dysbiosis Activate Peripheral T Cells With Autoimmune Behaviors in Glaucoma Patients

### Heat Shock Proteins in Stress Response

In glaucoma eyes, chronic stress challenges such as elevated IOP, oxidative stress, glutamate excitotoxicity, deprivation of neurotrophic factors, and ionic imbalance are considered to be primary triggers for neuroinflammation and RGC loss (19, 20). These stress stimuli can be sensed by multiple mechanical and nonmechanical stress receptors expressed on RGCs, including pannexin-1 (Panx1), P2X7 receptor (P2X7R), and transient receptor potential vanilloid isoform 4 (TRPV4), which leads to the production of danger signals (21). Heat shock proteins (HSPs) are one of such signal and have been recognized as principal autoantigens involved in the pathogenesis of glaucoma in both animal disease models and patients (22–25). HSPs are a group of highly conserved proteins that are ubiquitous in cellular organisms and are classified based on their molecular weights into 7 major families (small HSPs, HSP40, HSP60, HSP70, HSP90, HSP100, and HSP110) (26). Under physiological and stressful conditions, HSPs can serve as molecular chaperones to help refold misfolded proteins, enhance the survival of cells, and resist apoptosis (27, 28). Thus, HSPs are innate protectors of cells in the stress response. In addition, although most HSPs are constitutively intracellular components, under stress challenges, their expression is upregulated, and some HSPs can be released into the extracellular space to provoke immune reactions (29). Complex autoantibody patterns, including antibodies for small HSPs (HSP27, B-crystallin, and vimentin) and HSP70 in the aqueous humor have been detected in patients with various subtypes of glaucoma (30, 31). HSPs can induce both innate and adaptive immunity (32). In addition, a high local level of HSP27 itself is also found to be sufficiently pathogenic for glaucoma neural damage (33). However, it should be noted that HSPs should not be solely considered as proinflammatory factors but may induce immune suppression at suitable concentrations (34). Some HSPs also have neuroprotective effects. For example, induction of endogenous HSP72 in rats protects against neurodegeneration in acute IOP elevation (35, 36). Ongoing clinical trials are even applying HSPs to suppress the overactivation of the immune system in patients with rheumatoid arthritis and COVID-19 (37, 38). Thus, in patients with glaucoma, it is generally believed that aberrant production of HSPs under stress conditions and dysregulated autoimmune response in the long term, such as in the case of gut dysbiosis, may tilt the balance and result in uncontrolled neuroinflammation (39).

### Gut Microbiota Shapes the Immune System

Recently, researchers have revealed a potential link between gut dysbiosis and glaucoma, with the immune system as the connecting bridge. The gut microbiota is a critical factor that shapes the peripheral immune system and has been found to contribute to the activation of autoimmune T cells in glaucoma and other neurodegenerative diseases (40–42). The gut microbiota serves as the major source of bacterial HSPs in the human body, which cross-react with the highly conserved human HSPs to provoke autoimmunity and RGC damage (40, 43).

The pathological roles of gut dysbiosis in glaucoma are found to be chiefly mediated by T cells rather than autoantibodies or humoral immunity. In a mouse ocular hypertension (OHT) model induced by microbead injection, transient elevation of IOP resulted in a prolonged activation and infiltration of interferon-γ-secreting CD4-positive T cells in the RGC layer and subsequent neurodegeneration. The activated T cells were reactive to HSP27, which, along with its autoantibody, were found to increase in the serum after the OHT challenge. This prolonged autoimmunity to the retina, however, was absent in *TCRβ* ^-/-^ mice with deficit T cell immunity but not in *Igh6* ^-/-^ mice with deficit B cell immunity (40). On the other hand, although the infiltration of plasma cells in the retina and deposition of autoantibodies are also evident in the retina of glaucoma animal models, their causative roles in glaucoma remain elusive based on the finding that inhibition of B cells or autoantibody deprivation brings no significant benefits of RGC protection (44–46). Moreover, mice raised in a germ-free environment were found to be resistant to chronic OHT challenge and did not result in similar T cell infiltration in the retina, indicating the fundamental role of preexisting gut microbiota modulation of the immune system in the trigger of glaucomatous retinal damage (40).

**Figure 1:**
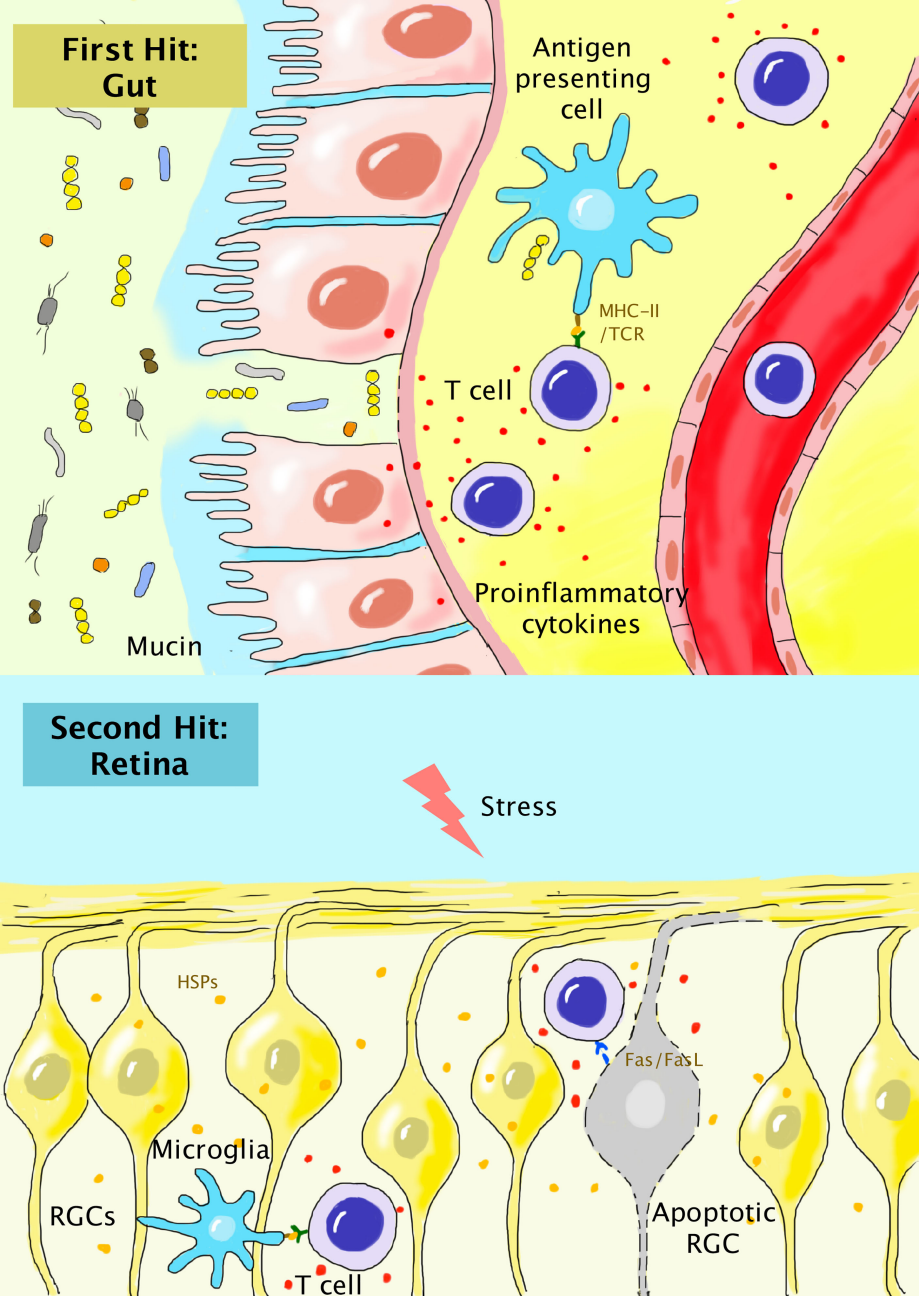
“Dual-hit hypothesis” for the association of peripherally-activated T cells during gut dysbiosis with glaucoma. In gut dysbiosis, the alteration of the components of gut microbiota, compromised mucin and gut epithelium, and dislocation of bacteria result in chronic local inflammation. Autoreactive T cells primed by microbial HSPs are generated and enter the systemic circulation (first hit). In the retina, the chronic stress response results in the release of HSPs and activation of residential microglia. Autoreactive T cells breach the compromised BRB and become reactivated in the retinal parenchyma (second hit). T cells can further induce the apoptosis of RGCs *via* the interaction of Fas/FasL.

The exact mechanisms of how gut microbiota shapes the host systemic immune system remain to be further elucidated, and the local gut mucosal immune system, as well as the compromised gut vascular barrier (GVB), have been suggested to play a role. Studies have found that primary open-angle glaucoma (POAG) patients have different compositions of gut microbiota compared to healthy individuals, with a higher abundance of *Prevotellaceae, Enterobacteriaceae, Escherichia coli*, and decreased number of *Megamonas* and *Bacteroides plebeius* (47). *Helicobacter pylori* infection was also linked to POAG and normal-tension glaucoma (NTG) in a recent meta-analysis (48). The opening of the GVB and dislocation of bacteria and bacterial elements not only affects autoimmune T cells, but may initially result in an overall chronic shift from the anti-inflammatory microenvironment to the pro-inflammatory one locally, systemically, and even at far target sites such as the brain and retina by activating the innate immune system (49). In a mouse experiment, a single peripheral administration of the bacterial element lipopolysaccharide (LPS) resulted in prolonged activation of brain resident microglial cells for over 10 months, resulting in progressive neurodegeneration (50). In the retina, resident microglia can recognize microbial pathogen-associated molecular pattern (PAMP) molecules *via* Toll-like receptors (TLRs), and upregulate the secretion of proinflammatory cytokines, including interleukin (IL)-6, IL-1β, and tumor necrosis factor-α (TNF-α), as well as major histocompatibility complex (MHC) II, an essential element for antigen presentation to T cells during peripheral bacterial infection (51, 52). The chronic activation of innate immunity in the retina, in turn, can facilitate the homing and infiltration of peripheral primed T cells into the retina. Studies have found that peripheral T cells responsive to microbial antigens in the intestine through autoreactive T cell receptors (TCRs) can break the blood-retinal barrier (BRB) and infiltrate the retina, which has previously been considered an immune-privileged site (53). This so-called “dual-hit hypothesis” (first hit in GVB and second hit in BRB) proposed by Braak et al. was first used to describe the potential role of gut dysbiosis in the pathogenesis of PD but is also supposed to be suitable for glaucoma and other neurodegenerative disorders (**Figure 1**). Interestingly, in patients with POAG, dislocation of gut *Helicobacter pylori* is even found in the trabecular meshwork (54). However, it is not clear whether the dislocation of gut bacteria itself is pathogenic or essential for glaucoma or is it just an epiphenomenon of GVB impairment. In addition, recent evidence also suggests that an imbalance in the oral microbiota may also contribute to the pathogenesis of glaucoma, which needs further investigation (55, 56). Above all, emerging evidence suggests that gut dysbiosis may result in the activation of both innate and adaptive immune systems. Through the cross-reaction of human and bacterial HSPs, peripherally-activated T cells may break the BRB and result in RGC damage under stressful conditions.

Recently, modulation of the gut microbiota to reshape the systemic immune response has become an emerging therapy for glaucoma. A study found the beneficial roles of fermented maize slurry and its supernatant rich in probiotic bacteria to reshape gut microbiota in rats in the modulation of retinal immune reaction and protection of RGCs (57). Further studies, especially clinical trials, to illuminate the crosstalk between the retina and gut may provide more valuable information on the pathogenesis of glaucoma and explore the possible intervention methods.

## Compromised BRB Is Essential for T Cell Infiltration in Glaucoma Eyes

The neural retina is a delicate visual transmission system and is firmly protected from the BRB, which controls the entrance of peripheral immune cells under normal conditions (58). Recently, some scholars have proposed the breakdown of BRB as an essential pathogenic step in glaucoma (59). This theory, although lacks definite evidence, is primarily supported by the observations of retinal T cell infiltration in different animal models of glaucoma (40, 60–62). In addition, the breakdown of blood-brain barrier (BBB) has been observed in some other CNS neurodegenerative diseases. Their common pathogenic features with glaucoma and the similar structures of the BBB and BRB also suggest the potential role of BRB breakdown in glaucoma development (13, 63–65).

The neural retina is isolated from the systemic circulation by the inner BRB (iBRB, formed with nonfenestrated retinal capillary endothelium, vascular basement membrane, pericytes, astrocytes end-feet, and microglial cells) and from the leaky choroidal vessels by the outer BRB (oBRB, formed with tightly connected RPE that stands on the Bruch’s membrane) (66). Acute IOP elevation is sufficient to alter the protein levels of tight junctions and adherens junctions of the RPE and subsequently affect the integrity of the BRB in glaucoma animal models (67). In dogs with primary glaucoma, disruption of PRE and extravasation of T cells and plasma proteins suggest the breakdown of both iBRB and oBRB (60). Acute induction of OHT in rats results in a significant reduction in pericyte coverage after 7-10 days, but the extent of vascular leakage remains unchanged (68). However, in patients with POAG and NTG, neurodegeneration is a slowly progressive event, and the breakdown of the BRB is likely associated with the long-standing retinal parainflammation otherwise. For example, aging as a contributing factor of glaucoma is associated with the progressive loss of BRB integrity and a low level of retinal inflammation (69). Some degenerative proteins such as amyloid-beta (Aβ) and hyperphosphorylated tau (p-tau), which are hallmarks of CNS neurodegenerative diseases are also found in the retina of glaucoma patients (14). These degenerative proteins contribute to the local inflammatory response and compromise BRB function by damaging tight junctions of the RPE and activating retinal microglial cells (70–72). The parainflammation state makes the retina vulnerable to the attack by adaptive autoimmune cells of peripheral origin.

The infiltration of T cells into retinal parenchyma includes 2 major steps: first, the extravasation from retinal vessels and then the breaching of glia limitans formed by astrocyte end-feet. The whole process is a continuous action that relies on the close interaction of T cells with multiple local factors and cells, as discussed in detail below.

### T Cell Extravasation

**Figure 2:**
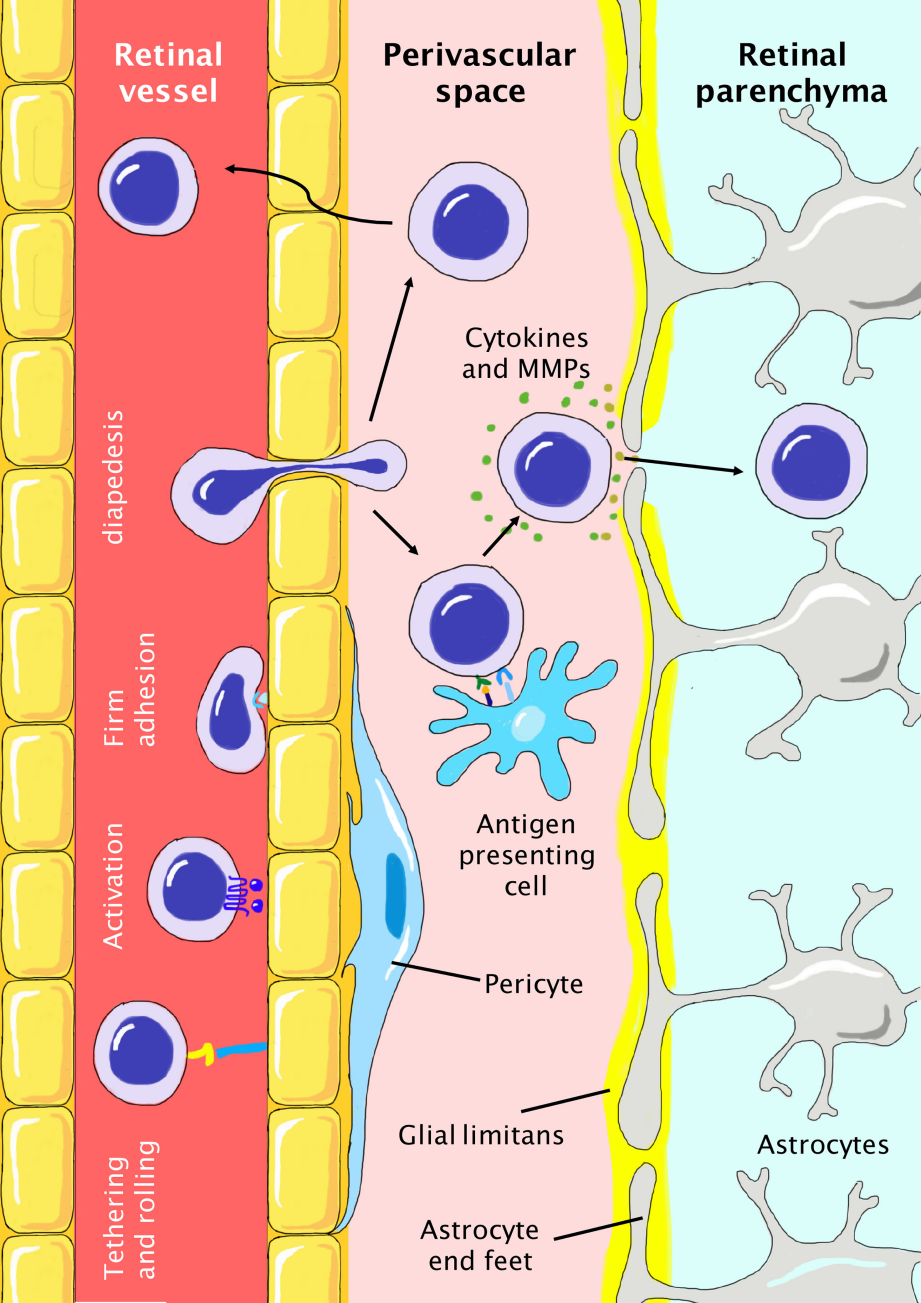
Infiltration of peripheral T cells into the retinal parenchyma during the pathogenesis of glaucoma. The BRB is tightly protected with nonfenestrated vascular endothelium, basement membrane, pericytes, and glia limitans formed by astrocyte end-feet. T cells need to first reach the perivascular space and then breach the glia limitans to finally infiltrate the retinal parenchyma. T cell extravasation includes steps of initial tethering and rolling, activation, firm adhesion, and final diapedesis. In the perivascular space, T cells need to be reactivated by antigen-presenting cells and secrete cytokines and MMPs to breach the glia limitans. Otherwise, T cells have to travel back to the vessel. In glaucoma, the local proinflammatory microenvironment favors the infiltration of T cells due to the higher abundance of molecules (cytokines, selectins, and chemokines) and cells (microglia, dendritic cells) involved in these processes.

As retinal capillaries are nonfenestrated, T cells have to escape through the tightly-connected vascular endothelium to reach the paravascular space in the retina (**Figure 2**). The extravasation of T cells into the retina is decided by the state of T lymphocyte activation, the state of retinal vascular endothelium, the microenvironment of the neuroretina, and local blood flow, which all affect the lymphocyte-endothelium interaction (73, 74). A study of T cell subsets in glaucoma patients revealed a significant shift in the T cell population and a greater stimulation response (75). When the BRB is intact and vascular endothelial cells are nonactive, peripherally-activated T lymphocytes can also cross the BRB and scan the retina for immune surveillance. Nevertheless, intravenous infusion of 5×10^6^ activated ovalbumin-specific T cells in rats only results in the transient opening of the BRB and activation of resident retinal microglial cells that subsides within 3 days (76). This indicates that intact and quiescent BRB can endure the challenge of systemic inflammation. However, when the retina is inflamed, activated vascular endothelium becomes highly adhesive for circulating lymphocytes by the upregulation of the expression of surface adhesion molecules, and a higher level of T cell extravasation is expected. The whole process of T cell trafficking across the retinal vascular endothelium is a complex action and includes 4 steps: tethering and rolling on the luminal surface, activation, firm adhesion on the vascular endothelium, and diapedesis (**Figure 2**) **(** 74). In the initial step, the interaction of selectins and integrins on the surface of lymphocytes and endothelial cells helps capture circulating immune cells to roll slowly and finally become arrested at the luminal surface (77). This initial attachment is not firm enough, and subsequent activation *via* chemokines presented by endothelial cells is needed to induce clustering and conformational changes of integrins to improve their affinity and avidity (78). T lymphocytes, now tightly grasped by endothelial cells, can break tight junctions and crawl out of blood vessels *via* transcellular or paracellular pathways (79–81).

In patients with glaucoma, the alterations of both T cell states and induced expression of local cytokines/chemokines may collectively contribute to the elevated extravasation of T cells. Some proinflammatory cytokines, particularly interferon-γ (IFN-γ), tumor necrotizing factor-α (TNF-α), and interleukin-1 (IL-1), are elevated in the aqueous humor or tears of glaucoma patients, indicating a globally proinflammatory state (82–84). These cytokines help T cell tethering and rolling by inducing the expression of integrins on the cell surface (85). Under resting conditions, only approximately 5% of lymphocytes derived from peripheral lymph nodes adhere to the retinal vascular endothelium, which doubles when the endothelium is activated by IFN-γ or IL-1 (86). In addition, peripherally activated T lymphocytes are also able to upregulate the expression of intercellular adhesion molecule-1 (ICAM-1) on the retina, a key integrin molecule for T cell rolling (87). In addition, RGCs of glaucomatous eyes demonstrate significantly elevated expression of genes involved in chemokine signaling (88). In the murine retina, the β chemokine and its receptor C-C chemokine ligand 5/C-C chemokine receptor type 5 (CCL5/CCR5) are constitutively expressed and can respond to IOP challenge (89). CCR5, which is inducible on activation, is found to be involved in the recruitment of T-helper 1 (Th1) cells into the mouse retina (90). Another pair of chemokines (C-X-C motif) ligand 10/(C-X-C motif) receptor 3 (CXCL10/CXCR3) are found to be induced by acute IOP elevation and subsequently contribute to the release of proinflammatory cytokines, elevated expression of E-selectin, and infiltration of inflammatory cells (91). In patients with glaucoma, the levels of chemokines of macrophage chemoattractant protein-1 (MCP-1), CXCR3, CCL2, and CCL7 show prognostic value and are correlated with disease progression (92–94). On the other hand, the induced expression of corresponding chemokines and receptors on the T cell surface under inflamed conditions favors their interplay with the vascular endothelium (90, 95). Overall, the activation of both T cells and the vascular endothelium are two arms of inflammatory episodes in glaucoma that promote T cell extravasation.

### The Breaching of Glia Limitans and Roles of Antigen-Presenting Cells

The retinal vessels are tightly wrapped with end-feet of astroglial cells that serve as a double barrier for T cell infiltration into the retinal parenchyma after extravasation (**Figure 2**). This physical barrier, named glia limitans, is concentric to the retinal vessels and creates a paravascular space. Tightening glia limitans with induced matrix metalloproteinase-3 attenuates lymphocyte infiltration after optic nerve injury (96). To cross the glia limitans, primed T cells need to be reactivated by the cognate antigen presented by antigen-presenting cells (APCs) or otherwise have to re-enter the retinal circulation. Thus, such APCs serve as major gatekeepers of the BRB, as they decide the fate of extravasated T cells. Compared with the CNS, the retina lacks meningeal layers and choroidal plexus which harbor numerous MHC-II^+^ APCs such as marginal dendritic cells and meningeal macrophages (97). In the pathogenesis of glaucoma, it is still under debate whether the APCs that initiate retinal inflammation are exogenous or activated resident cells. To serve as initial APCs, candidate cells need to distribute in the paravascular region and constitutively express MHC-II and costimulatory molecules. In a canine model of acute primary angle-closure glaucoma (PACG), infiltration of MHC-II^+^ phagocytes in the optic nerve and retina is observed within the first 24 hours, along with the infiltration of circulatory immune cells and RGC loss (98). However, it is controversial whether the infiltration of systemic phagocytes precedes the infiltration of T cells or *vice versa*. Some researchers also identified some innate players that may serve as initial APCs in the retina. A special group of microglial cells that are located in the paravascular space instead of retinal parenchyma are found to constitutively express high levels of CD45, MHC-I, and MHC-II (99). However, these CD45^+^ microglial cells demonstrate only a weak activation effect on primed T cells (100). Thus, the player(s) of initial antigen presentation and associated antigen in glaucoma remain to be determined, and their dynamic change and interplay with T cells during the disease course need to be further investigated. The initial antigen presentation process may serve as a novel therapeutic target to halt the initiation of neuroinflammation in glaucoma.

Currently, the study of BRB breakdown and the pathogenesis of glaucoma is still an emerging research field, and many detailed aspects remain to be determined. As suggested by studies of AD, the breakdown of the BBB seems to be one of the initiating events that contribute to the subsequent tissue deposition of degenerated proteins and immune cell infiltration (101). Acute animal glaucoma models suggest the temporary breakdown of the BRB in response to elevated IOP and a globally proinflammatory state that facilitates T cell infiltration. However, whether BRB breakdown only serves as the initial trigger of glaucoma neuroinflammation or is essential during the full term of disease progression remains to be determined by appropriate animal models and clinical studies. In addition, the spatial-temporal pattern of T infiltration in the glaucoma retina remains largely unknown.

## Retinal Gliosis and Interplay With T Cells

The retinal parenchyma harbors a group of endogenous immunocompetent cells including microglial cells, astrocytes, and Müller cells, and the latter two are also called macroglial cells. Under physical conditions, retinal glial cells closely interact with neurons and are highly versatile, participating in functions of mechanical support, metabolite transport, nourishment, tissue remodeling, and immunosurveillance (102). As key players of the retinal immune system, glial cells are distinct from conventional immune cells in the systemic circulation, as they are sequestered by the BRB and have no access to lymph nodes for lymphocyte priming. Nevertheless, the compromised BRB of glaucoma enables their contact with myeloid-derived peripheral immune cells that infiltrate the retina. These glial cells serve as sensors, mediators, and effectors of the immune response and are found to be early responders in the pathogenesis of glaucoma (103, 104).

### Different States of Retinal Glial Cells

**Figure 3:**
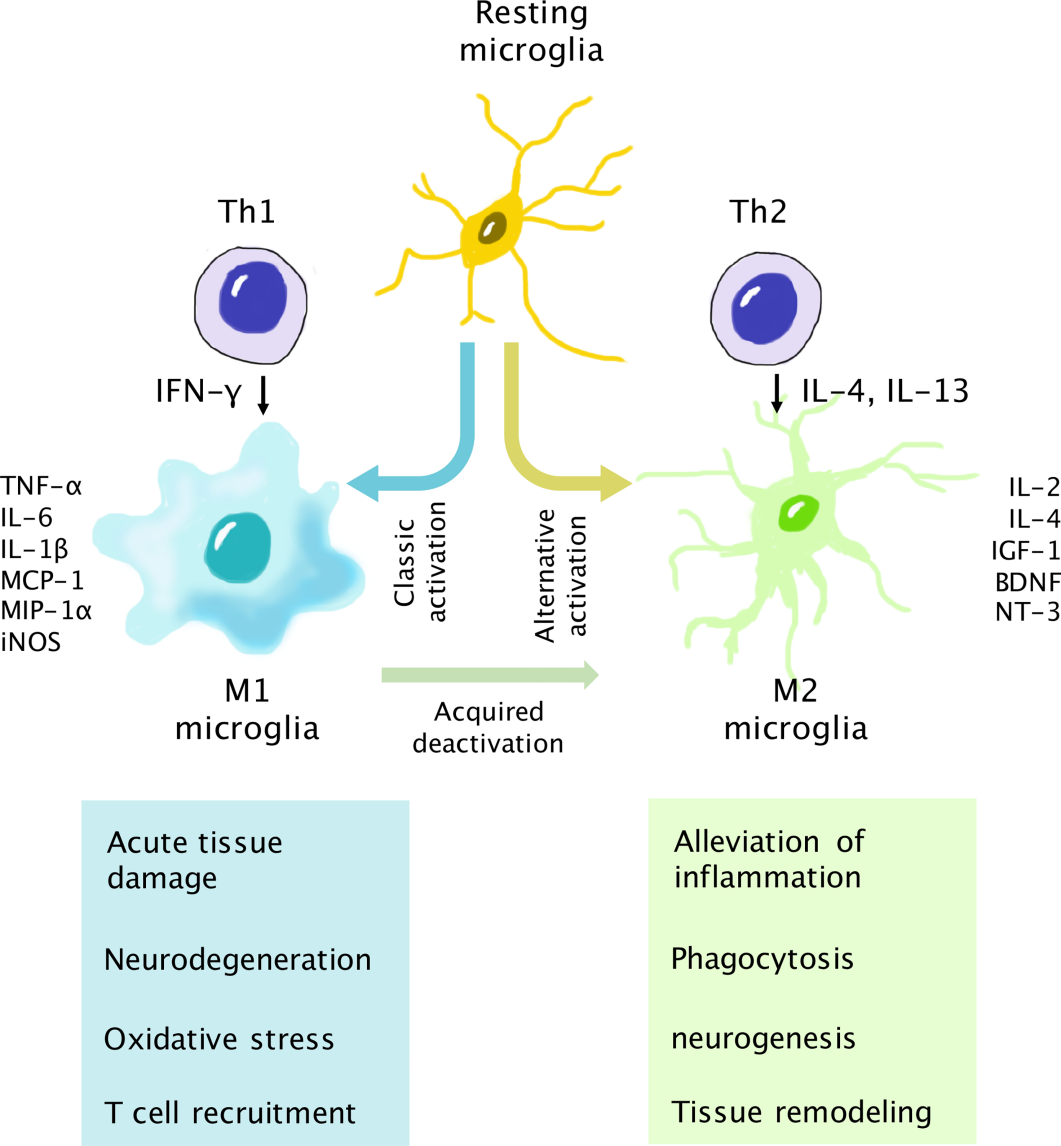
Interaction of T cells and microglia for neurodegeneration and neuroprotection. Upon activation, microglia assume either the M1 (neurotoxic) or M2 (neurotrophic) phenotype. The Th1 cytokine IFN-γ is associated with classic activation of the M1 phenotype. M1 microglia secrete proinflammatory cytokines, chemokines, and inducible nitric oxide synthase (iNOS). The Th1/M1 interaction results in the escalation of retinal inflammation and may lead to neurodegeneration if the reaction is not well controlled. In turn, the Th2 cytokines IL-4 and IL-13 are involved in alternative activation into the M2 phenotype, which secretes anti-inflammatory cytokines and neurotrophic factors. The Th2/M2 interaction results in the alleviation of inflammation and helps neuron survival. In addition, activated M1 microglia can transform into M2 microglia *via* the acquired deactivation process in the presence of IL-10 and TGF-β to control the overactivation of the immune response.

In glaucoma eyes, resident glial cells undergo a dynamic change with close coordination of each other in a time-dependent manner during the disease course (105, 106). This process, so-called “retinal gliosis” contributes to both neurodegeneration and neuroprotection, depending on the severity and chronicity of the reaction. Retinal microglial cells and astrocytes are highly heterogeneous and can be chiefly divided into 2 states after activation, i.e., the neurotoxic form (M1 and A1 phenotype, respectively) and neurotrophic form (M2 and A2 phenotype) (107, 108). For example, retinal microglial cells, as the major effector and APCs, assume an M1 phenotype with amoeboid morphology when stimulated by the proinflammatory cytokine interferon-γ (IFN-γ), and are able to release TNF-α, IL1-β, superoxides, proteases, and reactive oxygen species (ROS). On the other hand, when stimulated by IL-4, microglial cells turn into the M2 phenotype characterized by thin cell bodies with ramified processes. The M2 phenotype is associated with immunomodulatory cytokines such as IL-4, IL-10, IL-13, and TGF-β (**Figure 3**) (5, 109). Animal models of acute OHT suggest that a predominant M1/A1 type of microglial cells and astrocytes in the acute phase contributes to the early proinflammatory state, which may subside thereafter. The dynamic turnover and interplay between microglial and macroglial cells in glaucoma set the background of retinal inflammation, which is reviewed in detail by Zhao et al. (110).

### T Cell-Microglial Interplay for Neurotoxicity

In the acute phase, infiltrated T cells interact with neurotoxic glial cells to induce the escalation of inflammation. This process can result in neurotoxicity and damage to RGCs if not properly controlled. Once breaching the BRB, peripherally activated T cells can interact with microglial cells, which serve as the bridge between innate immunity and adaptive immunity. The roles of microglia are versatile. First, microglial cells act as early sensors of the retinal stress response *via* inherent Toll-like receptors (TLRs) on their surfaces (111). They are found to rapidly respond to extrinsic danger signals such as HSPs and oxidative stress and assume a predominant M1 phenotype within 24 hours after the induction of OHT (112–114). For example, extracellular HSP27 can activate TLRs and their downstream nuclear factor-kappa-light-chain-enhancer (NFκB) pathway in microglial cells, which subsequently promotes the release of proinflammatory cytokines (TNF-α, IL-6, and IL-1β) and chemokines (MCP-1, MCP-3, MIP-1α, MIP-1β) to facilitate the recruitment and activation of T cells (115–117). Knockout of TIR-domain-containing adapter-inducing interferon-β (TRIF), an adaptor molecule downstream of the TLR3 pathway, results in reduced microglial activation and preserves RGCs following damage (118). Recently, studies found that microglial cells can also propagate inflammatory signals in a paracrine way through the release of exosomes and activate remote microglial cells (119, 120). Another important function of retinal microglial cells is their role as APCs. In the retinal parenchyma, APCs are responsible for the reactivation of T cells with cognate antigens by the expression of MHC class II molecules and costimulatory molecules (CD40, CD86, B7, *etc.*), which can subsequently result in the clonal proliferation of T cells (121). In glaucoma eyes, activated microglial cells gain a stronger ability of antigen presentation and secretion of proinflammatory immune mediators, which further escalate retinal inflammation (122, 123). Conversely, T helper cells are also able to differentially modify the phenotypes of microglial cells *via* the secretion of proinflammatory or suppressive cytokines (124, 125). CD4^+^ Th1 cells secrete IFN-γ for M1 conversion, while the Th2 interferon IL-4 is associated with the M2 phenotype. Adoptive transfer of primed T cells from hereditary glaucoma mice leads to focal activation of Iba1^+^ microglial cells with amoeboid morphology that stand in close proximity to infiltrated T cells (126). In tyrosinase T cell receptor (TCR) transgenic mice that spontaneously develop glaucoma, marked infiltration of T cells is observed. T cells, through induction of effector cytokines, lead to a robust increase in glial fibrillary acidic protein^+^ (GFAP) glial cells that colocalize to T cells in the nerve fiber layer (61). Above all, as demonstrated in **Figure 3**, the interactions of T cells and retinal microglia are reciprocal and collectively shape the inflammatory background in glaucoma.

Both T cells and microglia can directly contribute to RGC loss, but there are also some distinctions in their mechanisms of action, and their relative contributions remain elusive. Histological studies reveal that the neuroinflammation characterized by retinal gliosis and T cell infiltration parallel ongoing neural degeneration spatially and temporally (68, 127, 128). Primed T cells after immunization with HSP27 and HSP60 can induce the apoptosis of retinal ganglion cells by secretion of fas-ligand (FasL) and upregulation of its receptor on RGCs (129). On the other hand, microglial cells can also contribute to RGC loss in other distinct ways such as pyroptosis and complement-mediated synaptic pruning (130). In glaucomatous eyes, both intrinsic and extrinsic apoptosis pathways of RGCs are involved in cell death (116).

### T Cell-Microglial Interplay for Neuroprotection

The role of the bidirectional interplay between T cells and microglial cells is multifaceted. Apart from neurotoxicity, it also enhances the clearance of dead neurons *via* phagocytosis, prevents escalation of inflammation, and sets the chance for subsequent tissue remodeling and neural repair, which may benefit the survival of remaining RGCs (131). Damaged neurons can release intracellular components after necrosis, such as DNA and nucleotides, which are highly proinflammatory and trigger the spread of inflammation. Activated microglial cells gain stronger mobility and phagocytic power and quickly move to the damaged site to isolate damaged neurons. As found in other CNS neurodegenerative diseases, tissue deposition of Aβ and p-tau is evident in glaucoma eyes (132, 133). These degenerative proteins are pathogenic and involved in progressive neuron death. In the CNS, Aβ can be taken up by activated microglial cells *via* phagocytosis and contribute to T cell activation (134, 135). In turn, primed T cells with Aβ immunization can also enhance the uptake and removal of Aβ in the brain and demonstrate beneficial effects (136, 137). IL-4 and IL-10 secreted mainly by Th2 cells are beneficial for the clearance of tissue deposition and debris by accelerating microglial phagocytic activities (138). In addition, activated retinal microglial cells and infiltrating macrophages secrete proinflammatory IL-6 and TNF-α to stimulate CD4^+^CD25^+^ T cells and suppress T cell-mediated cytotoxicity (139). Following the phase of intense attack, microglial cells, macroglial cells, and T cells coordinate to resolve immune reactions in the retina. In this phase, microglial cells convert from the proinflammatory M1 phenotype to the neuroprotective M2 phenotype and are able to secrete neurotrophic factors to promote neuronal survival. The retina, as an immune-privileged tissue, also possesses multiple negative regulatory circuits to restrict inflammation. For example, retinal microglial cells, as well as neurons and endothelial cells, were found to constitutively express CD200. Through interaction with the CD200 receptor, it attenuates the activation of myeloid cells (140). Microglial cells upregulate the expression of programmed death ligand-1 (PD-L1) at the peak phase of activation and suppress Th1 function *via* interaction with PD1 on their surfaces (141, 142).

Above all, it is generally accepted that the T cell-microglia interaction has both protective and destructive consequences and is related to the disease course. However, it is still under debate how to appropriately modify the immune process and benefit the survival of RGCs in glaucoma patients. Some animal studies in acute OHT explicitly support the beneficial role of neurons by suppressing microglial functions in different ways (143–146). However, this may not mimic the true chronic nature of glaucoma in most patients. It seems that this prompt immune reaction elicited by the stress response is at least beneficial in the restriction of neuroinflammation, but the longstanding insults of IOP fluctuation may lead to chronic activation of the microglia-T cell axis that exceeds the normal immunomodulation. As suggested by animal models of glaucoma, retinal gliosis is not only an early response but also persists in the chronic phase. Microglial cells remain activated after IOP returns to the normal level, which may potentially explain the ongoing neurodegeneration in patients after IOP has been controlled (147). Autopsy studies in postmortem glaucoma eyes may serve as direct evidence for marked retinal gliosis and infiltration of activated microglial cells in the optic nerve head (111, 148, 149). Glaucomatous eyes of humans also assume a chronic environment of neuroinflammation characterized by IgG autoantibody accumulation and increased levels of proinflammatory cytokines (45). In addition, from the point of view of T cells, there is a lasting imbalance of Th1/Th2 cells that tilts to the proinflammatory side in glaucoma patients (150, 151). These findings support the theory that chronic insults from endogenous (IOP, oxidative stress, *etc.*) and exogenous sources (gut dysbiosis, aging, *etc.*) lead to overactivation and disorder in immune regulation that turns the T cell-microglial axis into a predominant neurotoxic mode in the long term. Above all, this suggests the validity of immune modulation therapy in the treatment of glaucoma, as discussed in more detail in the section below.

## The Emerging Role of T Cell-Based Immune Modulation Therapy in the Treatment of Glaucoma - A Lesson From CNS Neurodegenerative Diseases

As discussed earlier, the immune reactions of T cells and microglia have both beneficial and detrimental effects on RGC survival, depending on whether they are properly evoked and regulated. In glaucoma, the chronic local proinflammatory microenvironment unfavorably shapes the immune reaction to the neurotoxic form and leads to progressive RGC loss. Considering the essential role of immune cells in tissue repair and maintenance of homeostasis, it is generally accepted that immune modulation, instead of simple immune suppression, is a valid option for glaucoma. The term “protective autoimmunity”, which refers to the beneficial roles of autoreactive T cells in the protection of neurons in the CNS, has been recognized for more than 2 decades (152, 153). This subsequently leads to the active exploration of T cell-based immune modulation therapy in glaucoma and other CNS neurodegenerative diseases.

### Protective Autoimmunity in the CNS and Retina

Under physical conditions, peripherally primed T cells that recognize self-antigens can patrol the retina and brain parenchyma over time. This immunosurveillance is not only helpful for immune detection but also plays a role in maintaining neural homeostasis by actively engaging in multiple physiological activities, including neurogenesis, regulating spatial learning and memory, and assisting neuron survival with neurotrophic factors (154). Deprivation of CNS-specific autoreactive T cells halts the normal development of the CNS system and is involved in cognitive impairment in animal models and exacerbation of CNS injury (155). On the other hand, accumulating evidence suggests that the T cell-mediated immune response may also provide beneficial effects in neuroprotection in the case of acute neural damage. In a rat model of optic nerve crush and contusive spinal cord injury, single low-dose γ-irradiation treatment induces the activation of proinflammatory T cells that ultimately leads to spontaneous recovery, which is absent in mice with T cell deficiency or transferred with regulatory T cells (Tregs) (11). A prior traumatic brain injury evoked protective autoimmunity that was found to prevent RGC loss when the contralateral optic nerve was crushed later in a rat model (10). These protective effects are chiefly dependent on neuron-specific autoimmune T cells, as evidenced by the finding that only transgenic T cells overexpressing the T cell receptor for the CNS-specific antigen of myelin basic protein (MBP) rather than the nonself antigen of ovalbumin demonstrate protective effects on RGC survival after optic nerve injury (156). Thus, the involvement of autoimmune T cells in the protection of neurons during acute CNS insult indicates the validity of the immune modulation approach for the treatment of glaucoma.

### Cop-1 Therapy

One investigated approach of immune modulation therapy in glaucoma is active immunization with a weak self-antigen that stimulates a moderate autoreactive T cell response. Autoimmune T cells are expected to regulate the immune response in a beneficial way and coordinate with local and other circulatory immune cells to boost faster tissue repair. Copolymer-1 (Cop-1, or glatiramer acetate), a synthesized analog of MBP, is a suitable candidate because it serves as a weak agonist of numerous self-antigens in the CNS. Immunization with Cop-1 has been tested in many CNS neurodegenerative diseases, and its commercial product Copaxone^®^ has been approved by the US Food and Drug Administration (FDA) for the treatment of MS (157). Glaucoma shares common aspects of pathogenesis with MS, including generation and tissue deposition of common autoantibodies (anti-MBP) and activation of local microglial cells (45, 158). Clinical examination with optical coherence tomography (OCT) also indicates a close relationship between retinal RGC damage and MS neuropathy (15). Thus, Cop-1 as an approved therapy for MS is also explored in glaucoma treatment. Coculture of Cop-1-stimulated T cells and retinal microglia results in their reciprocal activation and the release of insulin-like growth factor-1 (IGF-1), brain-derived neurotrophic factor (BDNF), TNF-α, and IL-10 *in vitro*, which favors RGC survival *in vitro* (159). Immunization with Cop-1 by subcutaneous injection in a rat glaucoma model results in elevated intraretinal T cell infiltration and prevention of RGC loss, which indicates that the induced T cell response is more protective than destructive (160). In addition, Cop-1 immunization has been delivered in combination with stem cell transplantation to rescue and replenish RGCs in animal glaucoma models. Cop-1 immunization induces a local favorable environment by balancing the levels of proinflammatory (IFN-γ) and anti-inflammatory (IL-4) cytokines and the secretion of neurotrophic factors (161). Activated T cells also release chemoattractants (such as MCP-1) to facilitate the recruitment of stem cells and progenitor cells to damaged sites (162, 163). The synergic action results in improved landing and survival of transplanted stem cells and alleviates nerve damage in glaucoma animal models (164, 165). Recently, the interim results of an ongoing double-masked clinical trial investigating Cop-1 immunization for the treatment of acute primary angle-closure glaucoma (PACG) have been disclosed (trial No. 01936129). Thirty-eight patients with PACG received either 2 subcutaneous injections of Cop-1 or the placebo without adjuvant, one within 24 hours of onset, and the other one week later. The patients in the Cop-1 group demonstrated improved mean deviation and a trend of a lower mean number of progressing points, but the retinal nerve fiber layer thickness showed no difference after 16 weeks. Due to the small sample sizes in both arms and high interpersonal variation, the researchers could not confirm the protective role of Cop-1 in PACG based on the current evidence (166). In addition, as no serum or immune examination test results were reported, whether the 2 subcutaneous injections of Cop-1 without adjuvant elicit an adequately strong and long-lasting immune response cannot be determined.

### Concerns and Further Considerations on Immune Modulation Therapy for Glaucoma

Although immune modulation therapy seems to be a reasonable treatment option for glaucoma and has been well explored in CNS neurodegenerative diseases, in clinical practice, many conditions need to be optimized. Safety is a chief concern. Immunization with R16, a self-peptide from interphotoreceptor retinoid-binding protein, in a mouse model of acute OHT led to neuron protection but induced monophasic autoimmune uveitis in a susceptible mouse strain (167). More experience from CNS clinical trials warns that the stimulation of unwanted detrimental immune responses could lead to adverse events. In a phase II clinical trial investigating the tolerability and efficacy of intramuscular injection of self-antigen Aβ42 with QS-21 as the adjuvant in patients with mild to moderate AD, subacute meningoencephalitis occurred in 6% of patients in the test arm, which led to the termination of the study (168). An aberrant Th1 cell response is speculated to be responsible for the adverse inflammatory response, as QS-21 is a strong inducer of Th1 and infiltration of active T cells was found in an autopsy study (169). Similar meningoencephalitis has been reported in amyloid precursor protein-transgenic (APP/Tg) mice that express limited IFN-γ, but not in other strains. The activation of Th1 cells in a proinflammatory environment and their interplay with microglial cells are causative of neuroinflammation (170). Moreover, the adoptive transfer of Aβ-activated Th1 cells, but not Th2 cells, in mice results in local microglial activation and exacerbation of AD in a mouse model (171). As the patients with CNS neurodegenerative diseases such as AD and glaucoma assume a generally proinflammatory state, the immune response with self-antigen vaccination should be carefully modified, as the induction of proinflammatory Th1 cells may further tilt the balance of immunity. In turn, a vaccination that induces a predominant Th2 response is preferred and helps restore Th1/Th2 imbalance (151, 172). In the study of AD, a dominant Th2-type response has been achieved by multiple means, such as applying Th2-inducing adjuvants (alum) (173), modifying the structures of the self-antigen (174), liposome coating (175), DNA vaccination (176), intranasal immunization (177), and transcutaneous delivery (178), but activation of Th1 cells cannot be fully eradicated and may still be considered a potential risk. Currently, the only safety data of immune modulation therapy in glaucoma patients come from the pilot clinical trial of the Cop-1 vaccine, which shows no other adverse events other than injection site pain (166). In a rat glaucoma model, systemic administration of Cop-1 with complete Freud’s adjuvant evokes a strong Th2 response and IL-4 secretion that peaks at 7 days and lasts for more than 31 days, with no obvious Th1 activation (179). In addition, as an old drug in MS that has been monitored for more than 2 decades, subcutaneous injection of Copaxone^®^ daily or three times per week is well tolerated, which is also suitable for children and pregnant women (180, 181). These results support the Cop-1 vaccine as a relatively safe therapy, but its long-term safety in glaucoma should be further elucidated.

Currently, Cop-1 vaccination is the only immune modulation therapy that has been tested in animals and patients with glaucoma, and more clinical data are expected to reflect its validity in patients. In CNS neurodegenerative diseases, many forms of passive or active immune modulation therapy have been explored, including self-antigen vaccination (182), DNA vaccination (183, 184), and transfer Treg cells (185, 186). The highly shared pathological and immunological features of glaucoma with other CNS neurodegenerative diseases support these therapies as rational options for glaucoma, which is worth further investigation. In addition, parallel changes in retinal neurons with the CNS have been observed in animals and patients on immune modulation therapy for CNS disorders (187, 188). However, the development and clinical translation of a mature immune modulation therapy for glaucoma are much more challenging than expected. Experience from clinical trials of AD and other CNS neurodegenerative diseases implicates numerous factors that may affect the efficacy and safety of immune modulation therapy, including antigens for immune induction, the time window of injection, frequency and amount of injection, drug delivery route, adjuvants, drug carriers, and individual factors (age, sex, *etc.*) (153). The development of a suitable regimen for a specific disease should be explored on a case-by-case basis. For example, Cop-1 immunization prior to or on the day of OHT damage significantly prevents RGC loss, while immunization 48 hours later generates no protection (189). In addition, a single Cop-1 immunization with complete Fraud’s adjuvant in a mouse acute OHT model is protective, but it dose not work for the optic nerve transection injury or amyotrophic lateral sclerosis (ALS) (190, 191). On the other hand, daily subcutaneous injection of Cop-1 is most effective for MS, but this regimen causes negative effects on the female mouse ALS model (191). Thus, a careful selection of regimens based on extensive animal and human studies is essential for an immune modulation therapy for glaucoma. Most importantly, researchers need to keep in mind that chronic neurodegeneration is multifactorial and highly complicated, and the efficacy observed on pathology may not translate into clinical improvement. For example, Aβ42 immunization in Alzheimer’s patients showed obvious benefits of prolonged plaque removal. Nevertheless, most of the patients progressed to Braak stage V-VI and developed severe dementia before death (192). Thus, a reliable clinical evaluation of drug efficacy is vital for immune modulation therapy of glaucoma and other neurodegenerative diseases.

Above all, the development of a mature immunomodulation therapy for glaucoma needs many more investigations on its safety and practical conditions. Special attention should be given to avoid unwanted exacerbation of the Th1-mediated response. In addition to Cop-1 immunization, more novel therapies, such as DNA vaccination and cell therapy, are also worthy of investigation in glaucoma.

## Summary and Conclusions

The perspectives on the pathogenesis of glaucoma are continuously updating, and debates are ongoing as new evidence from animal or clinical studies comes out. Although the research, diagnosis, and treatment focus primarily on IOP, T cell-mediated immune attack and its dynamic interplay with retinal microglial cells have been recognized to be the culprit of glaucoma. Researchers are attempting to shape the immune reaction to take advantage of its favorable neuroprotective effect. However, currently, no clear clinical evidence on the beneficial role of immunomodulation therapy in glaucoma patients has been illuminated on patients. Most importantly, signs of activation of autoimmunity and imbalance of immune reactions, including autoantibodies, a shift in the T cell subpopulation, and activation of retinal innate immunity, are clearly involved in the early phase of glaucoma. However, whether these clinical signs and associated biomarkers can assist the early diagnosis and primary prevention of glaucoma remains elusive. In addition, basic research on the autoimmune aspects of glaucoma is still a fresh field. Many theories of immune reactions in glaucoma basically come from findings from other CNS neurodegenerative diseases. In particular, the spatial-temporal breakdown of the BRB and retinal infiltration of peripheral immune cells during the chronic disease course in glaucoma patients are needed to illuminate the real contribution of T cells. As with other neurodegenerative diseases, a chronic animal disease model that mimics the true complexity and chronicity of glaucoma development in humans is lacking. Thus, findings from the acute OHT model may not reflect the whole picture, and more autopsy studies from human glaucoma samples may provide more valuable information. Moreover, the close overlap of glaucoma with other CNS neurodegenerative diseases indicates the need for a closer follow-up of patients in both ophthalmic and neurologic clinics. Their common pathogenesis in immune-mediated neurodegeneration indicates the possibility of their parallel progression and reaction to common therapies. Therefore, the ophthalmic follow-up of patients with CNS neurodegenerative disease going on trials of immunomodulation therapy may also provide valuable insights for glaucoma treatment.

## Author Contributions

Conception and design – LW and XW. Manuscript preparation – LW. Critical revisions – XW. All authors contributed to the article and approved the submitted version.

## Funding

This work was supported by grants from the Natural Science Foundation of China (No.82070954); the Applied Basic Research Programs of Science and Technology Commission Foundation of Sichuan Province (No. 2019YJ0016); the Innovative Spark Grant of Sichuan University (No.2018SCUH0062); 1·3·5 Project for Disciplines of Excellence–Clinical Research Incubation Project, West China Hospital, Sichuan University (No.2021HXFH057); the Science & Technology Department of Sichuan Province (China) Funding Project (No.2021YFS0221); and the Postdoctoral Research Funding of West China Hospital, Sichuan University, China (No.2020HXBH044).

## Conflict of Interest

The authors declare that the research was conducted in the absence of any commercial or financial relationships that could be construed as a potential conflict of interest.

## Publisher’s Note

All claims expressed in this article are solely those of the authors and do not necessarily represent those of their affiliated organizations, or those of the publisher, the editors and the reviewers. Any product that may be evaluated in this article, or claim that may be made by its manufacturer, is not guaranteed or endorsed by the publisher.

## References

[1] Tian BY Yu JN Yang XG. Progress in Glaucoma Retinal Ganglion Cells Injury. Int J Ophthalmol (2009) 9(1):118–20. doi: 10.3969/j.issn.1672-5123.2009.01.041

[2] Quigley HA Broman AT. The Number of People With Glaucoma Worldwide in 2010 and 2020. Br J Ophthalmol (2006) 90(3):262–7. doi: 10.1136/bjo.2005.081224 PMC1856963 16488940

[3] Weinreb RN Aung T Medeiros FA. The Pathophysiology and Treatment of Glaucoma: A Review. JAMA (2014) 311(18):1901–11. doi: 10.1001/jama.2014.3192 PMC4523637 24825645

[4] Greco A Rizzo MI De Virgilio A Gallo A Fusconi M de Vincentiis M. Emerging Concepts in Glaucoma and Review of the Literature. Am J Med (2016) 129(9):1000.e7–1000.e13. doi: 10.1016/j.amjmed.2016.03.038 27125182

[5] Wei X Cho KS Thee EF Jager MJ Chen DF. Neuroinflammation and Microglia in Glaucoma: Time for a Paradigm Shift. J Neurosci Res (2019) 97(1):70–6. doi: 10.1002/jnr.24256 PMC6239948 29775216

[6] Mélik Parsadaniantz S Réaux-le Goazigo A Sapienza A Habas C Baudouin C. Glaucoma: A Degenerative Optic Neuropathy Related to Neuroinflammation? Cells (2020) 9(3):1–14. doi: 10.3390/cells9030535 PMC7140467 32106630

[7] Geyer O Levo Y. Glaucoma is an Autoimmune Disease. Autoimmun Rev (2020) 19(6):102535. doi: 10.1016/j.autrev.2020.102535 32234407

[8] Wakefield D Wildner G. Is Glaucoma an Autoimmune Disease? Clin Trans Immunol (2020) 9(10):e1180. doi: 10.1002/cti2.1180 PMC7586712 33133597

[9] Von Thun Und Hohenstein-Blaul N Bell K Pfeiffer N Grus FH. Autoimmune Aspects in Glaucoma. Eur J Pharmacol (2016) 787:105–18. doi: 10.1016/j.ejphar.2016.04.031 27090926

[10] Ben Simon GJ Hovda DA Harris NG Gomez-Pinilla F Goldberg RA. Traumatic Brain Injury Induced Neuroprotection of Retinal Ganglion Cells to Optic Nerve Crush. J Neurotrauma (2006) 23(7):1072–82. doi: 10.1089/neu.2006.23.1072 16866620

[11] Kipnis J Avidan H Markovich Y Mizrahi T Hauben E Prigozhina TB. Low-Dose Gamma-Irradiation Promotes Survival of Injured Neurons in the Central Nervous System *via* Homeostasis-Driven Proliferation of T Cells. Eur J Neurosci (2004) 19(5):1191–8. doi: 10.1111/j.1460-9568.2004.03207.x 15016077

[12] Barış M Tezel G. Immunomodulation as a Neuroprotective Strategy for Glaucoma Treatment. Curr Ophthalmol Rep (2019) 7(2):160–9. doi: 10.1007/s40135-019-00212-1 PMC6662642 31360618

[13] Ashok A Singh N Chaudhary S Bellamkonda V Kritikos AE Wise AS. Retinal Degeneration and Alzheimer’s Disease: An Evolving Link. Int J Mol Sci (2020) 21(19):1–17. doi: 10.3390/ijms21197290 PMC7582766 33023198

[14] Ghiso JA Doudevski I Ritch R Rostagno AA. Alzheimer’s Disease and Glaucoma: Mechanistic Similarities and Differences. J Glaucoma (2013) 22 Suppl 5(0 5):S36–8. doi: 10.1097/IJG.0b013e3182934af6 PMC3955061 23733125

[15] Jones-Odeh E Hammond CJ. How Strong is the Relationship Between Glaucoma, the Retinal Nerve Fibre Layer, and Neurodegenerative Diseases Such as Alzheimer’s Disease and Multiple Sclerosis? Eye (London England) (2015) 29(10):1270–84. doi: 10.1038/eye.2015.158 PMC4815693 26337943

[16] Ramirez AI de Hoz R Salobrar-Garcia E Salazar JJ Rojas B Ajoy D. The Role of Microglia in Retinal Neurodegeneration: Alzheimer’s Disease, Parkinson, and Glaucoma. Front Aging Neurosci (2017) 9:214. doi: 10.3389/fnagi.2017.00214 28729832 PMC5498525

[17] Tang J Tang Y Yi I Chen DF. The Role of Commensal Microflora-Induced T Cell Responses in Glaucoma Neurodegeneration. Prog Brain Res (2020) 256(1):79–97. doi: 10.1016/bs.pbr.2020.06.002 32958216

[18] Campos-Acuña J Elgueta D Pacheco R. T-Cell-Driven Inflammation as a Mediator of the Gut-Brain Axis Involved in Parkinson’s Disease. Front Immunol (2019) 10:239. doi: 10.3389/fimmu.2019.00239 30828335 PMC6384270

[19] Alqawlaq S Flanagan JG Sivak JM. All Roads Lead to Glaucoma: Induced Retinal Injury Cascades Contribute to a Common Neurodegenerative Outcome. Exp eye Res (2019) 183:88–97. doi: 10.1016/j.exer.2018.11.005 30447198

[20] Mozaffarieh M Flammer J. New Insights in the Pathogenesis and Treatment of Normal Tension Glaucoma. Curr Opin Pharmacol (2013) 13(1):43–9. doi: 10.1016/j.coph.2012.10.001 23092679

[21] Križaj D Ryskamp DA Tian N Tezel G Mitchell CH Slepak VZ. From Mechanosensitivity to Inflammatory Responses: New Players in the Pathology of Glaucoma. Curr Eye Res (2014) 39(2):105–19. doi: 10.3109/02713683.2013.836541 PMC3946931 24144321

[22] Tsai T Grotegut P Reinehr S Joachim SC. Role of Heat Shock Proteins in Glaucoma. Int J Mol Sci (2019) 20(20):1–13. doi: 10.3390/ijms20205160 PMC6834184 31635205

[23] Lorenz K Beck S Keilani MM Wasielica-Poslednik J Pfeiffer N Grus FH. Course of Serum Autoantibodies in Patients After Acute Angle-Closure Glaucoma Attack. Clin Exp Ophthalmol (2017) 45(3):280–7. doi: 10.1111/ceo.12864 27758063

[24] Joachim SC Grus FH Kraft D White-Farrar K Barnes G Barbeck M. Complex Antibody Profile Changes in an Experimental Autoimmune Glaucoma Animal Model. Invest Ophthalmol Visual science (2009) 50(10):4734–42. doi: 10.1167/iovs.08-3144 19458332

[25] Joachim SC Wax MB Seidel P Pfeiffer N Grus FH. Enhanced Characterization of Serum Autoantibody Reactivity Following HSP 60 Immunization in a Rat Model of Experimental Autoimmune Glaucoma. Curr Eye Res (2010) 35(10):900–8. doi: 10.3109/02713683.2010.495829 20858111

[26] Tsan MF Gao B. Heat Shock Protein and Innate Immunity. Cell Mol Immunol (2004) 1(4):274–9. doi: 10.1046/j.1365-2249.2002.01759.x 16225770

[27] Van Den Ijssel PRLA Smulders RHPH De Jong WW Bloemendal H. α-Crystallin: Molecular Chaperone and Heat Shock Protein. Ophthalmic Res (1996) 28(SUPPL. 1):39–43. [Conference Paper]. doi: 10.1159/000267941 8727962

[28] Picard D. Heat-Shock Protein 90, a Chaperone for Folding and Regulation. Cell Mol Life Sci (2002) 59(10):1640–8. doi: 10.1007/PL00012491 PMC11337538 12475174

[29] Vallespí MG García I. Heat Shock Protein in Inflammation and Cancer. Biotecnologia Aplicada (2008) 25(3):199–215.

[30] Joachim SC Bruns K Lackner KJ Pfeiffer N Grus FH. Antibodies to Alpha B-Crystallin, Vimentin, and Heat Shock Protein 70 in Aqueous Humor of Patients With Normal Tension Glaucoma and IgG Antibody Patterns Against Retinal Antigen in Aqueous Humor. Curr Eye Res (2007) 32(6):501–9. doi: 10.1080/02713680701375183 17612966

[31] Joachim SC Wuenschig D Pfeiffer N Grus FH. IgG Antibody Patterns in Aqueous Humor of Patients With Primary Open Angle Glaucoma and Pseudoexfoliation Glaucoma. Mol Vision (2007) 13:1573–9. 17893658

[32] Reinehr S Kuehn S Casola C Koch D Stute G Grotegut P. HSP27 Immunization Reinforces AII Amacrine Cell and Synapse Damage Induced by S100 in an Autoimmune Glaucoma Model. Cell Tissue Res (2018) 371(2):237–49. doi: 10.1007/s00441-017-2710-0 29064077

[33] Grotegut P Kuehn S Dick HB Joachim SC. Destructive Effect of Intravitreal Heat Shock Protein 27 Application on Retinal Ganglion Cells and Neurofilament. Int J Mol Sci (2020) 21(2):1–20. doi: 10.3390/ijms21020549 PMC7014083 31952234

[34] Zininga T Ramatsui L Shonhai A. Heat Shock Proteins as Immunomodulants. Molecules (Basel Switzerland) (2018) 23(11):1–17. doi: 10.3390/molecules23112846 PMC6278532 30388847

[35] Zhang MH Zhou XM Cui JZ Wang KJ Feng Y Zhang HA. Neuroprotective Effects of Dexmedetomidine on Traumatic Brain Injury: Involvement of Neuronal Apoptosis and HSP70 Expression. Mol Med Rep (2018) 17(6):8079–86. doi: 10.3892/mmr.2018.8898 PMC5983975 29693126

[36] Qing G Duan X Jiang Y. Heat Shock Protein 72 Protects Retinal Ganglion Cells in Rat Model of Acute Glaucoma. Yan ke xue bao = Eye Science (2005) 21(3):163–8. 17162855

[37] Hernandez-Cedeño M Venegas-Rodriguez R Peña-Ruiz R Bequet-Romero M Santana-Sanchez R Penton-Arias E. CIGB-258, a Peptide Derived From Human Heat-Shock Protein 60, Decreases Hyperinflammation in COVID-19 Patients. Cell Stress Chaperones (2021) 26(3):515–25. doi: 10.1007/s12192-021-01197-2 PMC7904296 33629254

[38] Corrales O Hernández L Prada D Gómez J Reyes Y López AM. CIGB-814, an Altered Peptide Ligand Derived From Human Heat-Shock Protein 60, Decreases Anti-Cyclic Citrullinated Peptides Antibodies in Patients With Rheumatoid Arthritis. Clin Rheumatol (2019) 38(3):955–60. doi: 10.1007/s10067-018-4360-3 30415439

[39] Chen H Tian A Wu Y Li R Han R Xu X. HSP70 Expression Before and After Treatment and its Clinical Value in Patients With Acute Angle-Closure Glaucoma. Exp Ther Med (2021) 21(3):253. doi: 10.3892/etm.2021.9683 33603860 PMC7851605

[40] Chen H Cho KS Vu THK Shen CH Kaur M Chen G. Commensal Microflora-Induced T Cell Responses Mediate Progressive Neurodegeneration in Glaucoma. Nat Commun (2018) 9(1):3209. doi: 10.1038/s41467-018-05681-9 30097565 PMC6086830

[41] Pluta R Ułamek-Kozioł M Januszewski S Czuczwar SJ. Gut Microbiota and Pro/Prebiotics in Alzheimer’s Disease. Aging (2020) 12(6):5539–50. doi: 10.18632/aging.102930 PMC7138569 32191919

[42] Steinberg S Daniels J Hazan S. The Relationship Between Parkinson’s Disease and the Microbiome. Pract Gastroenterol (2020) 44(2):38–9.

[43] Tanaka T Yamakawa N Yamaguchi H Okada AA Konoeda Y Ogawa T. Common Ntigenicity Between Yersinia Enterocolitica-Derived Heat-Shock Protein and the Retina, and its Role in Uveitis. Ophthalmic Res (1996) 28(5):284–8. doi: 10.1159/000267916 8979276

[44] Gramlich OW Teister J Neumann M Tao X Beck S von Pein HD. Immune Response After Intermittent Minimally Invasive Intraocular Pressure Elevations in an Experimental Animal Model of Glaucoma. J Neuroinflamm (2016) 13(1):82. doi: 10.1186/s12974-016-0542-6 PMC4836145 27090083

[45] Gramlich OW Beck S von Thun Und Hohenstein-Blaul N Boehm N Ziegler A Vetter JM. Enhanced Insight Into the Autoimmune Component of Glaucoma: IgG Autoantibody Accumulation and Pro-Inflammatory Conditions in Human Glaucomatous Retina. PloS One (2013) 8(2):e57557. doi: 10.1371/journal.pone.0057557 23451242 PMC3581473

[46] Ding QJ Cook AC Dumitrescu AV Kuehn MH. Lack of Immunoglobulins Does Not Prevent C1q Binding to RGC and Does Not Alter the Progression of Experimental Glaucoma. Invest Ophthalmol Visual Sci (2012) 53(10):6370–7. doi: 10.1167/iovs.12-10442 PMC3465017 22918632

[47] Gong H Zhang S Li Q Zuo C Gao X Zheng B. Gut Microbiota Compositional Profile and Serum Metabolic Phenotype in Patients With Primary Open-Angle Glaucoma. Exp Eye Res (2020) 191:107921. doi: 10.1016/j.exer.2020.107921 31917963

[48] Doulberis M Papaefthymiou A Polyzos SA Bargiotas P Liatsos C Srivastava DS. Association Between Active Helicobacter Pylori Infection and Glaucoma: A Systematic Review and Meta-Analysis. Microorganisms (2020) 8(6):e216. doi: 10.3390/microorganisms8060894 32545826 PMC7355761

[49] Fung TC Olson CA Hsiao EY. Interactions Between the Microbiota, Immune and Nervous Systems in Health and Disease. Nat Neurosci (2017) 20(2):145–55. doi: 10.1038/nn.4476 PMC6960010 28092661

[50] Qin L Wu X Block ML Liu Y Breese GR Hong JS. Systemic LPS Causes Chronic Neuroinflammation and Progressive Neurodegeneration. Glia (2007) 55(5):453–62. doi: 10.1002/glia.20467 PMC2871685 17203472

[51] Matcovitch-Natan O Winter DR Giladi A Vargas Aguilar S Spinrad A Sarrazin S. Microglia Development Follows a Stepwise Program to Regulate Brain Homeostasis. Sci (New York NY) (2016) 353(6301):aad8670. doi: 10.1126/science.aad8670 27338705

[52] Maneu V Noailles A Gómez-Vicente V Carpena N Cuenca N Gil ML. Immunosuppression, Peripheral Inflammation and Invasive Infection From Endogenous Gut Microbiota Activate Retinal Microglia in Mouse Models. Microbiol Immunol (2016) 60(9):617–25. doi: 10.1111/1348-0421.12405 27466067

[53] Horai R Zárate-Bladés CR Dillenburg-Pilla P Chen J Kielczewski JL Silver PB. Microbiota-Dependent Activation of an Autoreactive T Cell Receptor Provokes Autoimmunity in an Immunologically Privileged Site. Immunity (2015) 43(2):343–53. doi: 10.1016/j.immuni.2015.07.014 PMC4544742 26287682

[54] Zavos C Kountouras J Sakkias G Venizelos I Deretzi G Arapoglou S. Histological Presence of Helicobacter Pylori Bacteria in the Trabeculum and Iris of Patients With Primary Open-Angle Glaucoma. Ophthalmic Res (2012) 47(3):150–6. doi: 10.1159/000330053 22094712

[55] Astafurov K Elhawy E Ren L Dong CQ Igboin C Hyman L. Oral Microbiome Link to Neurodegeneration in Glaucoma. PloS One (2014) 9(9):e104416. doi: 10.1371/journal.pone.0104416 25180891 PMC4152129

[56] Pasquale LR Hyman L Wiggs JL Rosner BA Joshipura K McEvoy M. Prospective Study of Oral Health and Risk of Primary Open-Angle Glaucoma in Men: Data From the Health Professionals Follow-Up Study. Ophthalmology (2016) 123(11):2318–27. doi: 10.1016/j.ophtha.2016.07.014 PMC5077693 27554035

[57] Fafure AA Edem EE Obisesan AO Enye LA Adekeye AO Adetunji AE. Fermented Maize Slurry (Ogi) and Its Supernatant (Omidun) Mitigate Elevated Intraocular Pressure by Modulating BDNF Expression and Glial Plasticity in the Retina-Gut Axis of Glaucomatous Rats. J Complementary Integr Med (2021). doi: 10.1515/jcim-2021-0114 34380184

[58] Stein-Streilein J. Mechanisms of Immune Privilege in the Posterior Eye. Int Rev Immunol (2013) 32(1):42–56. doi: 10.3109/08830185.2012.740535 23360157

[59] Yang X Yu XW Zhang DD Fan ZG. Blood-Retinal Barrier as a Converging Pivot in Understanding the Initiation and Development of Retinal Diseases. Chin Med J (2020) 133(21):2586–94. doi: 10.1097/cm9.0000000000001015 PMC7722606 32852382

[60] Mangan BG Al-Yahya K Chen CT Gionfriddo JR Powell CC Dubielzig RR. Retinal Pigment Epithelial Damage, Breakdown of the Blood-Retinal Barrier, and Retinal Inflammation in Dogs With Primary Glaucoma. Vet Ophthalmol (2007) 10(Suppl 1):117–24. doi: 10.1111/j.1463-5224.2007.00585.x 17973843

[61] Husain S Abdul Y Webster C Chatterjee S Kesarwani P Mehrotra S. Interferon-Gamma (IFN-γ)-Mediated Retinal Ganglion Cell Death in Human Tyrosinase T Cell Receptor Transgenic Mouse. PloS One (2014) 9(2):e89392. doi: 10.1371/journal.pone.0089392 24586745 PMC3938457

[62] Gramlich OW Anderson MG Ding Q Kuehn MH. Adaptive Immune Responses in Glaucoma Promote IOP-Independent RCG Loss. Invest Ophthalmol Visual Sci (2015) 56(7):1694.

[63] Shi H Koronyo Y Fuchs DT Sheyn J Wawrowsky K Lahiri S. Retinal Capillary Degeneration and Blood-Retinal Barrier Disruption in Murine Models of Alzheimer’s Disease. Acta Neuropathologica Commun (2020) 8(1):202. doi: 10.1186/s40478-020-01076-4 PMC7686701 33228786

[64] Zhang M Zhong L Han X Xiong G Xu D Zhang S. Brain and Retinal Abnormalities in the 5xfad Mouse Model of Alzheimer’s Disease at Early Stages. Front Neurosci (2021) 15:681831. doi: 10.3389/fnins.2021.681831 34366774 PMC8343228

[65] Yoon SP Grewal DS Thompson AC Polascik BW Dunn C Burke JR. Retinal Microvascular and Neurodegenerative Changes in Alzheimer’s Disease and Mild Cognitive Impairment Compared With Control Participants. Ophthalmol Retina (2019) 3(6):489–99. doi: 10.1016/j.oret.2019.02.002 PMC6586560 31174670

[66] Runkle EA Antonetti DA. The Blood-Retinal Barrier: Structure and Functional Significance. Methods Mol Biol (Clifton NJ) (2011) 686:133–48. doi: 10.1007/978-1-60761-938-3_5 21082369

[67] Brockhaus K Melkonyan H Prokosch-Willing V Liu H Thanos S. Alterations in Tight- and Adherens-Junction Proteins Related to Glaucoma Mimicked in the Organotypically Cultivated Mouse Retina Under Elevated Pressure. Invest Ophthalmol Visual Sci (2020) 61(3):46. doi: 10.1167/iovs.61.3.46 PMC7401456 32207812

[68] Trost A Motloch K Bruckner D Schroedl F Bogner B Kaser-Eichberger A. Time-Dependent Retinal Ganglion Cell Loss, Microglial Activation and Blood-Retina-Barrier Tightness in an Acute Model of Ocular Hypertension. Exp Eye Res (2015) 136:59–71. doi: 10.1016/j.exer.2015.05.010 26001526

[69] Xu H Chen M Forrester JV. Para-Inflammation in the Aging Retina. Prog Retinal Eye Res (2009) 28(5):348–68. doi: 10.1016/j.preteyeres.2009.06.001 19560552

[70] Park SW Kim JH Park SM Moon M Lee KH Park KH. RAGE Mediated Intracellular Aβ Uptake Contributes to the Breakdown of Tight Junction in Retinal Pigment Epithelium. Oncotarget (2015) 6(34):35263–73. doi: 10.18632/oncotarget.5894 PMC4742103 26431165

[71] Walsh DT Montero RM Bresciani LG Jen AY Leclercq PD Saunders D. Amyloid-Beta Peptide Is Toxic to Neurons *In Vivo via* Indirect Mechanisms. Neurobiol Dis (2002) 10(1):20–7. doi: 10.1006/nbdi.2002.0485 12079400

[72] Anderson PJ Watts H Hille C Philpott K Clark P Gentleman MC. Glial and Endothelial Blood-Retinal Barrier Responses to Amyloid-Beta in the Neural Retina of the Rat. Clin Ophthalmol (Auckland NZ) (2008) 2(4):801–16. doi: 10.2147/opth.s3967 PMC2699783 19668434

[73] Mesri M Liversidge J Forrester JV. ICAM-1/LFA-1 Interactions in T-Lymphocyte Activation and Adhesion to Cells of the Blood-Retina Barrier in the Rat. Immunology (1994) 83(1):52–7. PMC1415018 7821966

[74] Crane IJ Liversidge J. Mechanisms of Leukocyte Migration Across the Blood-Retina Barrier. Semin Immunopathol (2008) 30(2):165–77. doi: 10.1007/s00281-008-0106-7 PMC2315689 18305941

[75] Yang X Zeng Q Göktas E Gopal K Al-Aswad L Blumberg DM. T-Lymphocyte Subset Distribution and Activity in Patients With Glaucoma. Invest Ophthalmol Visual Science (2019) 60(4):877–88. doi: 10.1167/iovs.18-26129 PMC6397017 30821813

[76] Hu P Pollard JD Chan-Ling T. Breakdown of the Blood-Retinal Barrier Induced by Activated T Cells of Nonneural Specificity. Am J Pathol (2000) 156(4):1139–49. doi: 10.1016/s0002-9440(10)64982-6 PMC1876898 10751337

[77] Liversidge J Sewell HF Forrester JV. Interactions Between Lymphocytes and Cells of the Blood-Retina Barrier: Mechanisms of T Lymphocyte Adhesion to Human Retinal Capillary Endothelial Cells and Retinal Pigment Epithelial Cells *In Vitro* . Immunology (1990) 71(3):390–6. PMC1384438 1980120

[78] Alon R Feigelson S. From Rolling to Arrest on Blood Vessels: Leukocyte Tap Dancing on Endothelial Integrin Ligands and Chemokines at Sub-Second Contacts. Semin Immunol (2002) 14(2):93–104. doi: 10.1006/smim.2001.0346 11978081

[79] Filippi MD. Neutrophil Transendothelial Migration: Updates and New Perspectives. Blood (2019) 133(20):2149–58. doi: 10.1182/blood-2018-12-844605 PMC6524565 30898863

[80] Greenwood J Calder VL. Lymphocyte Migration Through Cultured Endothelial Cell Monolayers Derived From the Blood-Retinal Barrier. Immunology (1993) 80(3):401–6. PMC1422218 8288317

[81] Filippi MD. Mechanism of Diapedesis: Importance of the Transcellular Route. Adv Immunol (2016) 129:25–53. doi: 10.1016/bs.ai.2015.09.001 26791857 PMC4889131

[82] Burgos-Blasco B Vidal-Villegas B Saenz-Frances F Morales-Fernandez L Perucho-Gonzalez L Garcia-Feijoo J. Tear and Aqueous Humour Cytokine Profile in Primary Open-Angle Glaucoma. Acta Ophthalmologica (2020) 98(6):e768–72. doi: 10.1111/aos.14374 32043817

[83] Huang P Qi Y Xu YS Liu J Liao D Zhang SS. Serum Cytokine Alteration is Associated With Optic Neuropathy in Human Primary Open Angle Glaucoma. J Glaucoma (2010) 19(5):324–30. doi: 10.1097/IJG.0b013e3181b4cac7 19730118

[84] Chua J Vania M Cheung CM Ang M Chee SP Yang H. Expression Profile of Inflammatory Cytokines in Aqueous From Glaucomatous Eyes. Mol Vision (2012) 18:431–8. PMC3283212 22355254

[85] Smith-Garvin JE Koretzky GA Jordan MS. T Cell Activation. Annu Rev Immunol (2009) 27:591–619. doi: 10.1146/annurev.immunol.021908.132706 19132916 PMC2740335

[86] Wang YF Calder VL Greenwood J Lightman SL. Lymphocyte Adhesion to Cultured Endothelial Cells of the Blood-Retinal Barrier. J Neuroimmunol (1993) 48(2):161–8. doi: 10.1016/0165-5728(93)90188-5 8227314

[87] Xu H Manivannan A Liversidge J Sharp PF Forrester JV Crane IJ. Requirements for Passage of T Lymphocytes Across non-Inflamed Retinal Microvessels. J Neuroimmunol (2003) 142(1-2):47–57. doi: 10.1016/s0165-5728(03)00258-3 14512163

[88] Wang DY Ray A Rodgers K Ergorul C Hyman BT Huang W. Global Gene Expression Changes in Rat Retinal Ganglion Cells in Experimental Glaucoma. Invest Ophthalmol Visual Sci (2010) 51(8):4084–95. doi: 10.1167/iovs.09-4864 PMC2910641 20335623

[89] Duncan DS McLaughlin WM Vasilakes N Echevarria FD Formichella CR Sappington RM. Constitutive and Stress-Induced Expression of CCL5 Machinery in Rodent Retina. J Clin Cell Immunol (2017) 8(3):1–29. doi: 10.4172/2155-9899.1000506 PMC5604884 28936366

[90] Crane IJ Xu H Wallace C Manivannan A Mack M Liversidge J. Involvement of CCR5 in the Passage of Th1-Type Cells Across the Blood-Retina Barrier in Experimental Autoimmune Uveitis. J Leukocyte Biol (2006) 79(3):435–43. doi: 10.1189/jlb.0305130 16365158

[91] Ha Y Liu H Xu Z Yokota H Narayanan SP Lemtalsi T. Endoplasmic Reticulum Stress-Regulated CXCR3 Pathway Mediates Inflammation and Neuronal Injury in Acute Glaucoma. Cell Death Dis (2015) 6(10):e1900. doi: 10.1038/cddis.2015.281 26448323 PMC4632306

[92] Siwak M Maślankiewicz M Nowak-Zduńczyk A Rozpędek W Wojtczak R Szymanek K. The Relationship Between HDAC6, CXCR3, and SIRT1 Genes Expression Levels With Progression of Primary Open-Angle Glaucoma. Ophthalmic Genet (2018) 39(3):325–31. doi: 10.1080/13816810.2018.1432061 29384425

[93] Lee NY Kim MH Park CK. Visual Field Progression is Associated With Systemic Concentration of Macrophage Chemoattractant Protein-1 in Normal-Tension Glaucoma. Curr Eye Res (2017) 42(7):1002–6. doi: 10.1080/02713683.2016.1276193 28306361

[94] Gao X Huang W Zhang X Du S Wang J Wang W. Chemokine (C-C Motif) Ligand 2 and Chemokine (C-C Motif) Ligand 7 in Angle-Closure Glaucoma. Acta Ophthalmologica (2016) 94(3):e220–4. doi: 10.1111/aos.12696 25726969

[95] Man SM Ma YR Shang DS Zhao WD Li B Guo DW. Peripheral T Cells Overexpress MIP-1alpha to Enhance Its Transendothelial Migration in Alzheimer’s Disease. Neurobiol Aging (2007) 28(4):485–96. doi: 10.1016/j.neurobiolaging.2006.02.013 16600437

[96] Lefevere E Salinas-Navarro M Andries L Noterdaeme L Etienne I Van Wonterghem E. Tightening the Retinal Glia Limitans Attenuates Neuroinflammation After Optic Nerve Injury. Glia (2020) 68(12):2643–60. doi: 10.1002/glia.23875 32645232

[97] Mundt S Greter M Flügel A Becher B. The CNS Immune Landscape From the Viewpoint of a T Cell. Trends Neurosciences (2019) 42(10):667–79. doi: 10.1016/j.tins.2019.07.008 31474310

[98] Scott EM Boursiquot N Beltran WA Dubielzig RR. Early Histopathologic Changes in the Retina and Optic Nerve in Canine Primary Angle-Closure Glaucoma. Vet Ophthalmol (2013) 16(Suppl 1):79–86. doi: 10.1111/vop.12046 23826772

[99] Provis JM Penfold PL Edwards AJ van Driel D. Human Retinal Microglia: Expression of Immune Markers and Relationship to the Glia Limitans. Glia (1995) 14(4):243–56. doi: 10.1002/glia.440140402 8530182

[100] Gregerson DS Sam TN McPherson SW. The Antigen-Presenting Activity of Fresh, Adult Parenchymal Microglia and Perivascular Cells From Retina. J Immunol (Baltimore Md 1950) (2004) 172(11):6587–97. doi: 10.4049/jimmunol.172.11.6587 15153473

[101] Yamazaki Y Kanekiyo T. Blood-Brain Barrier Dysfunction and the Pathogenesis of Alzheimer’s Disease. Int J Mol Sci (2017) 18(9):1–19. doi: 10.3390/ijms18091965 PMC5618614 28902142

[102] Vecino E Rodriguez FD Ruzafa N Pereiro X Sharma SC. Glia-Neuron Interactions in the Mammalian Retina. Prog Retinal Eye Res (2016) 51:1–40. doi: 10.1016/j.preteyeres.2015.06.003 26113209

[103] Ramírez AI De Hoz R Rojas B Salazar JJ Salobrar-García E Vidal-Sanz M. Bilateral Early Activation of Macroglial Retinal Cells in a Mouse Model of Unilateral Laser-Induced Experimental Glaucoma. Glia (2017) 65:E104. doi: 10.1002/glia.23157

[104] Bosco A Steele MR Vetter ML. Early Microglia Activation in a Mouse Model of Chronic Glaucoma. J Comp Neurol (2011) 519(4):599–620. doi: 10.1002/cne.22516 21246546 PMC4169989

[105] Wang M Wang X Zhao L Ma W Rodriguez IR Fariss RN. Macroglia-Microglia Interactions *via* TSPO Signaling Regulates Microglial Activation in the Mouse Retina. J Neurosci Off J Soc Neurosci (2014) 34(10):3793–806. doi: 10.1523/jneurosci.3153-13.2014 PMC3942591 24599476

[106] Ellis-Behnke RG Jonas RA Jonas JB. The Microglial System in the Eye and Brain in Response to Stimuli *In Vivo* . J Glaucoma (2013) 22(Suppl 5):S32–5. doi: 10.1097/IJG.0b013e3182934aca 23733124

[107] Tang Y Le W. Differential Roles of M1 and M2 Microglia in Neurodegenerative Diseases. Mol Neurobiol (2016) 53(2):1181–94. doi: 10.1007/s12035-014-9070-5 25598354

[108] Fan YY Huo J. A1/A2 Astrocytes in Central Nervous System Injuries and Diseases: Angels or Devils? Neurochem Int (2021) 148:1–20. doi: 10.1016/j.neuint.2021.105080 34048845

[109] Varnum MM Ikezu T. The Classification of Microglial Activation Phenotypes on Neurodegeneration and Regeneration in Alzheimer’s Disease Brain. Archivum Immunologiae Therapiae Experimentalis (2012) 60(4):251–66. doi: 10.1007/s00005-012-0181-2 PMC4429536 22710659

[110] Zhao X Sun R Luo X Wang F Sun X. The Interaction Between Microglia and Macroglia in Glaucoma. Front Neurosci (2021) 15:610788. doi: 10.3389/fnins.2021.610788 34121982 PMC8193936

[111] Luo C Yang X Kain AD Powell DW Kuehn MH Tezel G. Glaucomatous Tissue Stress and the Regulation of Immune Response Through Glial Toll-Like Receptor Signaling. Invest Ophthalmol Visual Sci (2010) 51(11):5697–707. doi: 10.1167/iovs.10-5407 PMC3061506 20538986

[112] de Hoz R Ramírez AI González-Martín R Ajoy D Rojas B Salobrar-Garcia E. Bilateral Early Activation of Retinal Microglial Cells in a Mouse Model of Unilateral Laser-Induced Experimental Ocular Hypertension. Exp Eye Res (2018) 171:12–29. doi: 10.1016/j.exer.2018.03.006 29526796

[113] Ramírez AI Salazar JJ de Hoz R Rojas B Gallego BI Salobrar-García E. Macro- and Microglial Responses in the Fellow Eyes Contralateral to Glaucomatous Eyes. Prog Brain Res (2015) 220:155–72. doi: 10.1016/bs.pbr.2015.05.003 26497789

[114] Ramírez AI de Hoz R Fernández-Albarral JA Salobrar-Garcia E Rojas B Valiente-Soriano FJ. Time Course of Bilateral Microglial Activation in a Mouse Model of Laser-Induced Glaucoma. Sci Rep (2020) 10(1):4890. doi: 10.1038/s41598-020-61848-9 32184450 PMC7078298

[115] Lawrence T. The Nuclear Factor NF-kappaB Pathway in Inflammation. Cold Spring Harbor Perspect Biol (2009) 1(6):a001651. doi: 10.1101/cshperspect.a001651 PMC2882124 20457564

[116] Grotegut P Hoerdemann PJ Reinehr S Gupta N Dick HB Joachim SC. Heat Shock Protein 27 Injection Leads to Caspase Activation in the Visual Pathway and Retinal T-Cell Response. Int J Mol Sci (2021) 22(2):1–19. doi: 10.3390/ijms22020513 PMC7825587 33419223

[117] Zeng HY Zhu XA Zhang C Yang LP Wu LM Tso MO. Identification of Sequential Events and Factors Associated With Microglial Activation, Migration, and Cytotoxicity in Retinal Degeneration in Rd Mice. Invest Ophthalmol Visual Sci (2005) 46(8):2992–9. doi: 10.1167/iovs.05-0118 16043876

[118] Lin S Liang Y Zhang J Bian C Zhou H Guo Q. Microglial TIR-Domain-Containing Adapter-Inducing Interferon-β (TRIF) Deficiency Promotes Retinal Ganglion Cell Survival and Axon Regeneration *via* Nuclear Factor-κb. J Neuroinflamm (2012) 9:39. doi: 10.1186/1742-2094-9-39 PMC3471332 22361049

[119] Aires ID Ribeiro-Rodrigues T Boia R Catarino S Girão H Ambrósio AF. Exosomes Derived From Microglia Exposed to Elevated Pressure Amplify the Neuroinflammatory Response in Retinal Cells. Glia (2020) 68(12):2705–24. doi: 10.1002/glia.23880 32645245

[120] Aires ID Santiago AR. Microglial Exosomes in Retinal Neuroinflammation: Focus in Glaucoma. Neural Regeneration Res (2021) 16(9):1801–2. doi: 10.4103/1673-5374.306084 PMC8328767 33510084

[121] Tezel G Yang X Luo C Peng Y Sun SL Sun D. Mechanisms of Immune System Activation in Glaucoma: Oxidative Stress-Stimulated Antigen Presentation by the Retina and Optic Nerve Head Glia. Invest Ophthalmol Visual Sci (2007) 48(2):705–14. doi: 10.1167/iovs.06-0810 PMC2494942 17251469

[122] Baudouin C Kolko M Melik-Parsadaniantz S Messmer EM. Inflammation in Glaucoma: From the Back to the Front of the Eye, and Beyond. Prog Retinal Eye Res (2021) 83:100916. doi: 10.1016/j.preteyeres.2020.100916 33075485

[123] Tezel G Chauhan BC LeBlanc RP Wax MB. Immunohistochemical Assessment of the Glial Mitogen-Activated Protein Kinase Activation in Glaucoma. Invest Ophthalmol Visual Sci (2003) 44(7):3025–33. doi: 10.1167/iovs.02-1136 12824248

[124] Matsubara T Pararajasegaram G Wu GS Rao NA. Retinal Microglia Differentially Express Phenotypic Markers of Antigen-Presenting Cells *In Vitro* . Invest Ophthalmol Visual Sci (1999) 40(13):3186–93. 10586941

[125] Schoenborn JR Wilson CB. Regulation of Interferon-Gamma During Innate and Adaptive Immune Responses. Adv Immunol (2007) 96:41–101. doi: 10.1016/s0065-2776(07)96002-2 17981204

[126] Gramlich OW Ding QJ Zhu W Cook A Anderson MG Kuehn MH. Adoptive Transfer of Immune Cells From Glaucomatous Mice Provokes Retinal Ganglion Cell Loss in Recipients. Acta Neuropathologica Commun (2015) 3:56. doi: 10.1186/s40478-015-0234-y PMC4591529 26374513

[127] Chidlow G Ebneter A Wood JP Casson RJ. Evidence Supporting an Association Between Expression of Major Histocompatibility Complex II by Microglia and Optic Nerve Degeneration During Experimental Glaucoma. J Glaucoma (2016) 25(8):681–91. doi: 10.1097/ijg.0000000000000447 27253969

[128] Naskar R Wissing M Thanos S. Detection of Early Neuron Degeneration and Accompanying Microglial Responses in the Retina of a Rat Model of Glaucoma. Invest Ophthalmol Visual Sci (2002) 43(9):2962–8. 12202516

[129] Wax MB Tezel G Yang J Peng G Patil RV Agarwal N. Induced Autoimmunity to Heat Shock Proteins Elicits Glaucomatous Loss of Retinal Ganglion Cell Neurons *via* Activated T-Cell-Derived Fas-Ligand. J Neurosci Off J Soc Neurosci (2008) 28(46):12085–96. doi: 10.1523/jneurosci.3200-08.2008 PMC2683273 19005073

[130] Chen H Deng Y Gan X Li Y Huang W Lu L. NLRP12 Collaborates With NLRP3 and NLRC4 to Promote Pyroptosis Inducing Ganglion Cell Death of Acute Glaucoma. Mol Neurodegeneration (2020) 15(1):26. doi: 10.1186/s13024-020-00372-w PMC7161290 32295623

[131] Shaked I Porat Z Gersner R Kipnis J Schwartz M. Early Activation of Microglia as Antigen-Presenting Cells Correlates With T Cell-Mediated Protection and Repair of the Injured Central Nervous System. J Neuroimmunol (2004) 146(1-2):84–93. doi: 10.1016/j.jneuroim.2003.10.049 14698850

[132] Janciauskiene S Westin K Grip O Krakau T. Detection of Alzheimer Peptides and Chemokines in the Aqueous Humor [Article]. Eur J Ophthalmol (2011) 21(1):104–11. doi: 10.5301/EJO.2010.2108 20602326

[133] Wang L Mao X. Role of Retinal Amyloid-β in Neurodegenerative Diseases: Overlapping Mechanisms and Emerging Clinical Applications. Int J Mol Sci (2021) 22(5):1–25. doi: 10.3390/ijms22052360 PMC7956232 33653000

[134] Schetters STT Gomez-Nicola D Garcia-Vallejo JJ Van Kooyk Y. Neuroinflammation: Microglia and T Cells Get Ready to Tango. Front Immunol (2017) 8:1905. doi: 10.3389/fimmu.2017.01905 29422891 PMC5788906

[135] Das R Chinnathambi S. Microglial Priming of Antigen Presentation and Adaptive Stimulation in Alzheimer’s Disease. Cell Mol Life Sci CMLS (2019) 76(19):3681–94. doi: 10.1007/s00018-019-03132-2 PMC11105582 31093687

[136] Nicoll JA Wilkinson D Holmes C Steart P Markham H Weller RO. Neuropathology of Human Alzheimer Disease After Immunization With Amyloid-Beta Peptide: A Case Report. Nat Med (2003) 9(4):448–52. doi: 10.1038/nm840 12640446

[137] Fisher Y Nemirovsky A Baron R Monsonego A. T Cells Specifically Targeted to Amyloid Plaques Enhance Plaque Clearance in a Mouse Model of Alzheimer’s Disease. PloS One (2010) 5(5):e10830. doi: 10.1371/journal.pone.0010830 20520819 PMC2877087

[138] Koenigsknecht-Talboo J Landreth GE. Microglial Phagocytosis Induced by Fibrillar Beta-Amyloid and IgGs are Differentially Regulated by Proinflammatory Cytokines. J Neurosci Off J Soc Neurosci (2005) 25(36):8240–9. doi: 10.1523/jneurosci.1808-05.2005 PMC6725530 16148231

[139] Geng Y Lu Z Guan J van Rooijen N Zhi Y. Microglia/Macrophages and CD4(+)CD25(+) T Cells Enhance the Ability of Injury-Activated Lymphocytes to Reduce Traumatic Optic Neuropathy *In Vitro* . Front Immunol (2021) 12:687898. doi: 10.3389/fimmu.2021.687898 34484185 PMC8414969

[140] Dick AD Carter D Robertson M Broderick C Hughes E Forrester JV. Control of Myeloid Activity During Retinal Inflammation. J Leukocyte Biol (2003) 74(2):161–6. doi: 10.1189/jlb.1102535 12885931

[141] Hu J He H Yang Z Zhu G Kang L Jing X. Programmed Death Ligand-1 on Microglia Regulates Th1 Differentiation *via* Nitric Oxide in Experimental Autoimmune Encephalomyelitis. Neurosci Bullet (2016) 32(1):70–82. doi: 10.1007/s12264-015-0010-9 PMC5563746 26769487

[142] Schreiner B Bailey SL Shin T Chen L Miller SD. PD-1 Ligands Expressed on Myeloid-Derived APC in the CNS Regulate T-Cell Responses in EAE. Eur J Immunol (2008) 38(10):2706–17. doi: 10.1002/eji.200838137 PMC2727707 18825752

[143] Harun-Or-Rashid M Inman DM. Reduced AMPK Activation and Increased HCAR Activation Drive Anti-Inflammatory Response and Neuroprotection in Glaucoma. J Neuroinflamm (2018) 15(1):313. doi: 10.1186/s12974-018-1346-7 PMC6234605 30424795

[144] Aires ID Boia R Rodrigues-Neves AC Madeira MH Marques C Ambrósio AF. Blockade of Microglial Adenosine A(2A) Receptor Suppresses Elevated Pressure-Induced Inflammation, Oxidative Stress, and Cell Death in Retinal Cells. Glia (2019) 67(5):896–914. doi: 10.1002/glia.23579 30667095 PMC6590475

[145] Ferreira-Silva J Aires ID Boia R Ambrósio AF Santiago AR. Activation of Adenosine A(3) Receptor Inhibits Microglia Reactivity Elicited by Elevated Pressure. Int J Mol Sci (2020) 21(19):1–15. doi: 10.3390/ijms21197218 PMC7582754 33007835

[146] Wang K Peng B Lin B. Fractalkine Receptor Regulates Microglial Neurotoxicity in an Experimental Mouse Glaucoma Model. Glia (2014) 62(12):1943–54. doi: 10.1002/glia.22715 24989686

[147] Ebneter A Casson RJ Wood JP Chidlow G. Microglial Activation in the Visual Pathway in Experimental Glaucoma: Spatiotemporal Characterization and Correlation With Axonal Injury. Invest Ophthalmol Visual Sci (2010) 51(12):6448–60. doi: 10.1167/iovs.10-5284 20688732

[148] Yuan L Neufeld AH. Activated Microglia in the Human Glaucomatous Optic Nerve Head. J Neurosci Res (2001) 64(5):523–32. doi: 10.1002/jnr.1104 11391707

[149] Yang X Luo C Cai J Powell DW Yu D Kuehn MH. Neurodegenerative and Inflammatory Pathway Components Linked to TNF-α/TNFR1 Signaling in the Glaucomatous Human Retina. Invest Ophthalmol Visual Sci (2011) 52(11):8442–54. doi: 10.1167/iovs.11-8152 PMC3208177 21917936

[150] Guo C Wu N Niu X Wu Y Chen D Guo W. Comparison of T Helper Cell Patterns in Primary Open-Angle Glaucoma and Normal-Pressure Glaucoma. Med Sci Monitor Int Med J Exp Clin Res (2018) 24:1988–96. doi: 10.12659/msm.904923 PMC5900463 29616680

[151] Wong M Huang P Li W Li Y Zhang SS Zhang C. T-Helper1/T-Helper2 Cytokine Imbalance in the Iris of Patients With Glaucoma. PloS One (2015) 10(3):e0122184. doi: 10.1371/journal.pone.0122184 25811482 PMC4374700

[152] Schwartz M Ziv Y. Immunity to Self and Self-Maintenance: A Unified Theory of Brain Pathologies. Trends Immunol (2008) 29(5):211–9. doi: 10.1016/j.it.2008.01.003 18328784

[153] Schwartz M London A Shechter R. Boosting T-Cell Immunity as a Therapeutic Approach for Neurodegenerative Conditions: The Role of Innate Immunity. Neuroscience (2009) 158(3):1133–42. doi: 10.1016/j.neuroscience.2008.12.013 19103265

[154] Graber JJ Dhib-Jalbut S. Protective Autoimmunity in the Nervous System. Pharmacol Ther (2009) 121(2):147–59. doi: 10.1016/j.pharmthera.2008.10.001 19000712

[155] Herz J Köster C Crasmöller M Abberger H Hansen W Felderhoff-Müser U. Peripheral T Cell Depletion by FTY720 Exacerbates Hypoxic-Ischemic Brain Injury in Neonatal Mice. Front Immunol (2018) 9:1696. doi: 10.3389/fimmu.2018.01696 30127782 PMC6087766

[156] Yoles E Hauben E Palgi O Agranov E Gothilf A Cohen A. Protective Autoimmunity is a Physiological Response to CNS Trauma. J Neurosci Off J Soc Neurosci (2001) 21(11):3740–8. doi: 10.1523/jneurosci.21-11-03740.2001 PMC6762728 11356861

[157] Rück I Bartholomes T. Copaxone® - A New Dimension in Multiple Sclerosis Therapy. Pharmazie Unserer Zeit (2002) 31(1):115–7. doi: 10.1002/1615-1003(200201)31:1<115::AID-PAUZ115>3.0.CO;2-A German. copaxone.

[158] Choi S Guo L Cordeiro MF. Retinal and Brain Microglia in Multiple Sclerosis and Neurodegeneration. Cells (2021) 10(6):1–21. doi: 10.3390/cells10061507 PMC8232741 34203793

[159] Qian S Tang Y Cheng L Sun X Tian J Zhou C. Interaction of Copolymer-1-Activated T Cells and Microglia in Retinal Ganglion Cell Protection. Clin Exp Ophthalmol (2013) 41(9):881–90. doi: 10.1111/ceo.12110 23566072

[160] Li X Qian SH Sun XH. [Protection of Autoimmunity Induced by Copolymer-1 on Optic Nerve: Experiment With Rat Glaucoma Models]. Zhonghua yi xue za zhi (2008) 88(30):2152–4. doi: 10.3321/j.issn:0376-2491.2008.30.016 19080481

[161] Zhou X Xia XB. Retinal Stem Cells Transplantation Combined With Copolymer-1 Immunization Reduces Interferon-Gamma Levels in an Experimental Model of Glaucoma. Int J Ophthalmol (2011) 4(6):594–8. doi: 10.3980/j.issn.2222-3959.2011.06.04 PMC3340800 22553727

[162] Belmadani A Tran PB Ren D Miller RJ. Chemokines Regulate the Migration of Neural Progenitors to Sites of Neuroinflammation. J Neurosci Off J Soc Neurosci (2006) 26(12):3182–91. doi: 10.1523/jneurosci.0156-06.2006 PMC2740990 16554469

[163] Ziv Y Avidan H Pluchino S Martino G Schwartz M. Synergy Between Immune Cells and Adult Neural Stem/Progenitor Cells Promotes Functional Recovery From Spinal Cord Injury. Proc Natl Acad Sci USA (2006) 103(35):13174–9. doi: 10.1073/pnas.0603747103 PMC1559772 16938843

[164] Fu WC Jiang Y Zhang L. Effect of RSCs Combined With COP-1 on Optic Nerve Damage in Glaucoma Rat Model. Asian Pacific J Trop Med (2014) 7(4):317–20. doi: 10.1016/s1995-7645(14)60047-x 24507684

[165] Zhou X Xia XB Xiong SQ. Neuro-Protection of Retinal Stem Cells Transplantation Combined With Copolymer-1 Immunization in a Rat Model of Glaucoma. Mol Cell Neurosci (2013) 54:1–8. doi: 10.1016/j.mcn.2012.12.001 23246669

[166] Fan KR Baskaran M Nongpiur ME Htoon HM de Leon JMS Perera SA. Investigating the Neuroprotective Effect of Copolymer-1 in Acute Primary Angle Closure - Interim Report of a Randomized Placebo-Controlled Double-Masked Clinical Trial. Acta Ophthalmologica (2019) 97(6):e827–32. doi: 10.1111/aos.14099 30916898

[167] Bakalash S Kessler A Mizrahi T Nussenblatt R Schwartz M. Antigenic Specificity of Immunoprotective Therapeutic Vaccination for Glaucoma. Invest Ophthalmol Visual Sci (2003) 44(8):3374–81. doi: 10.1167/iovs.03-0080 12882784

[168] Orgogozo JM Gilman S Dartigues JF Laurent B Puel M Kirby LC. Subacute Meningoencephalitis in a Subset of Patients With AD After Abeta42 Immunization. Neurology (2003) 61(1):46–54. doi: 10.1212/01.wnl.0000073623.84147.a8 12847155

[169] Ferrer I Boada Rovira M Sánchez Guerra ML Rey MJ Costa-Jussá F. Neuropathology and Pathogenesis of Encephalitis Following Amyloid-Beta Immunization in Alzheimer’s Disease. Brain Pathol (Zurich Switzerland) (2004) 14(1):11–20. doi: 10.1111/j.1750-3639.2004.tb00493.x PMC8095815 14997933

[170] Monsonego A Imitola J Petrovic S Zota V Nemirovsky A Baron R. Abeta-Induced Meningoencephalitis is IFN-Gamma-Dependent and Is Associated With T Cell-Dependent Clearance of Abeta in a Mouse Model of Alzheimer’s Disease. Proc Natl Acad Sci USA (2006) 103(13):5048–53. doi: 10.1073/pnas.0506209103 PMC1458792 16549802

[171] Browne TC McQuillan K McManus RM O’Reilly JA Mills KH Lynch MA. IFN-γ Production by Amyloid β-Specific Th1 Cells Promotes Microglial Activation and Increases Plaque Burden in a Mouse Model of Alzheimer’s Disease. J Immunol (Baltimore Md 1950) (2013) 190(5):2241–51. doi: 10.4049/jimmunol.1200947 23365075

[172] Cao C Arendash GW Dickson A Mamcarz MB Lin X Ethell DW. Abeta-Specific Th2 Cells Provide Cognitive and Pathological Benefits to Alzheimer’s Mice Without Infiltrating the CNS. Neurobiol Dis (2009) 34(1):63–70. doi: 10.1016/j.nbd.2008.12.015 19167499 PMC5546306

[173] Cribbs DH Ghochikyan A Vasilevko V Tran M Petrushina I Sadzikava N. Adjuvant-Dependent Modulation of Th1 and Th2 Responses to Immunization With Beta-Amyloid. Int Immunol (2003) 15(4):505–14. doi: 10.1093/intimm/dxg049 PMC1483061 12663680

[174] Ghochikyan A Mkrtichyan M Petrushina I Movsesyan N Karapetyan A Cribbs DH. Prototype Alzheimer’s Disease Epitope Vaccine Induced Strong Th2-Type Anti-Abeta Antibody Response With Alum to Quil A Adjuvant Switch. Vaccine (2006) 24(13):2275–82. doi: 10.1016/j.vaccine.2005.11.039 PMC2081151 16368167

[175] Muhs A Hickman DT Pihlgren M Chuard N Giriens V Meerschman C. Liposomal Vaccines With Conformation-Specific Amyloid Peptide Antigens Define Immune Response and Efficacy in APP Transgenic Mice. Proc Natl Acad Sci USA (2007) 104(23):9810–5. doi: 10.1073/pnas.0703137104 PMC1887581 17517595

[176] Matsumoto Y Kohyama K. Development of a New DNA Vaccine for Alzheimer Disease Targeting Abeta Species and Amyloiditogenic Peptides in the Brain. Brain Pathol (2010) 20:16. doi: 10.1111/j.1750-3639.2010.00420.x

[177] Weiner HL Lemere CA Maron R Spooner ET Grenfell TJ Mori C. Nasal Administration of Amyloid-Beta Peptide Decreases Cerebral Amyloid Burden in a Mouse Model of Alzheimer’s Disease. Ann Neurol (2000) 48(4):567–79. doi: 10.1002/1531-8249(200010)48:4<567::AID-ANA3>3.0.CO;2-W 11026440

[178] Nikolic WV Bai Y Obregon D Hou H Mori T Zeng J. Transcutaneous Beta-Amyloid Immunization Reduces Cerebral Beta-Amyloid Deposits Without T Cell Infiltration and Microhemorrhage. Proc Natl Acad Sci USA (2007) 104(7):2507–12. doi: 10.1073/pnas.0609377104 PMC1892920 17264212

[179] Cheng LN Qian SH Sun XH Tian J Tang YT Wang Y. [Expressional Changes of Th1 and Th2 Cells in Retina of a Rat Glaucoma Model Vaccinated by Cop-1]. Zhonghua yi xue za zhi (2011) 91(39):2789–92. doi: 10.3760/cma.j.issn.0376-2491.2011.39.016 22322063

[180] Boster AL Ford CC Neudorfer O Gilgun-Sherki Y. Glatiramer Acetate: Long-Term Safety and Efficacy in Relapsing-Remitting Multiple Sclerosis. Expert Rev Neurotherapeutics (2015) 15(6):575–86. doi: 10.1586/14737175.2015.1040768 25924547

[181] Ziemssen T Ashtamker N Rubinchick S Knappertz V Comi G. Long-Term Safety and Tolerability of Glatiramer Acetate 20 Mg/Ml in the Treatment of Relapsing Forms of Multiple Sclerosis. Expert Opin Drug Safety (2017) 16(2):247–55. doi: 10.1080/14740338.2017.1274728 27989217

[182] NovakPZilkaNZilkovaMKovacechBSkrabanaROndrusM. AADvac1, an Active Immunotherapy for Alzheimer’s Disease and Non Alzheimer Tauopathies: An Overview of Preclinical and Clinical Development. J Prev Alzheimer’s Dis (2019) 6(1):63–9. doi: 10.14283/jpad.2018.45 30569088

[183] DaSilva KA Brown ME McLaurin J. Reduced Oligomeric and Vascular Amyloid-β Following Immunization of TgCRND8 Mice With an Alzheimer’s DNA Vaccine. Vaccine (2009) 27(9):1365–76. doi: 10.1016/j.vaccine.2008.12.044 19150380

[184] Evans CF Davtyan H Petrushina I Hovakimyan A Davtyan A Hannaman D. Epitope-Based DNA Vaccine for Alzheimer’s Disease: Translational Study in Macaques. Alzheimer’s Dementia (2014) 10(3):284–95. doi: 10.1016/j.jalz.2013.04.505 PMC3825833 23916838

[185] Thonhoff JR Beers DR Zhao W Pleitez M Simpson EP Berry JD. Expanded Autologous Regulatory T-Lymphocyte Infusions in ALS: A Phase I, First-in-Human Study. Neurology(R) Neuroimmunol Neuroinflamm (2018) 5(4):e465. doi: 10.1212/nxi.0000000000000465 PMC5961523 29845093

[186] Baek H Ye M Kang GH Lee C Lee G Choi DB. Neuroprotective Effects of CD4+CD25+Foxp3+ Regulatory T Cells in a 3xtg-AD Alzheimer’s Disease Model. Oncotarget (2016) 7(43):69347–57. doi: 10.18632/oncotarget.12469 PMC5342482 27713140

[187] Doustar J Rentsendorj A Torbati T Regis GC Fuchs DT Sheyn J. Parallels Between Retinal and Brain Pathology and Response to Immunotherapy in Old, Late-Stage Alzheimer’s Disease Mouse Models. Aging Cell (2020) 19(11):e13246. doi: 10.1111/acel.13246 PMC7681044 33090673

[188] Lambe J Risher H Filippatou AG Murphy OC Sotirchos ES Ehrhardt H. Modulation of Retinal Atrophy With Rituximab in Multiple Sclerosis. Neurology (2021) 96(20):e2525–33. doi: 10.1212/wnl.0000000000011933 PMC8205480 33827962

[189] Schori H Kipnis J Yoles E WoldeMussie E Ruiz G Wheeler LA. Vaccination for Protection of Retinal Ganglion Cells Against Death From Glutamate Cytotoxicity and Ocular Hypertension: Implications for Glaucoma. Proc Natl Acad Sci USA (2001) 98(6):3398–403. doi: 10.1073/pnas.041609498 PMC30665 11248090

[190] Blair M Pease ME Hammond J Valenta D Kielczewski J Levkovitch-Verbin H. Effect of Glatiramer Acetate on Primary and Secondary Degeneration of Retinal Ganglion Cells in the Rat. Invest Ophthalmol Visual Sci (2005) 46(3):884–90. doi: 10.1167/iovs.04-0731 15728544

[191] Schwartz M Bukshpan S Kunis G. Application of Glatiramer Acetate to Neurodegenerative Diseases Beyond Multiple Sclerosis: The Need for Disease-Specific Approaches. BioDrugs Clin Immunotherapeutics Biopharmaceuticals Gene Ther (2008) 22(5):293–9. doi: 10.2165/00063030-200822050-00002 18778111

[192] Wisniewski T. Follow-Up of Active Aβ Immunization in Alzheimer Disease. Nat Rev Neurology (2019) 15(9):495–6. doi: 10.1038/s41582-019-0239-4 31308505

